# A mean-field model of neural networks with PV and SOM interneurons reveals connectivity-based mechanisms of gamma oscillations

**DOI:** 10.1371/journal.pcbi.1014378

**Published:** 2026-06-10

**Authors:** Farzin Tahvili, Martin Vinck, Matteo Di Volo

**Affiliations:** 1 Université Lyon 1, INSERM, SBRI, U1208, Lyon, France; 2 Donders Centre for Neuroscience, Department of Neurophysics, Radboud University Nijmegen, Nijmegen, Netherlands; Research Centre Jülich: Forschungszentrum Jülich GmbH, GERMANY

## Abstract

Classic theoretical models of cortical oscillations are based on the interactions between two populations of excitatory and inhibitory neurons. Nevertheless, experimental studies and network simulations suggest that interneuron subclasses such as parvalbumin (PV) and somatostatin (SOM) exert distinct control over oscillatory dynamics. Yet, we lack a theoretical understanding of the mechanisms underlying oscillations in E-PV-SOM circuits and of the differences with respect to the classical mechanisms for oscillations in simpler E–I networks. Here, we derive a biologically realistic mean-field model of a canonical three-population E-PV-SOM circuit. This model robustly generates oscillations whose features are consistent with experimental observations, including the relative timing of PV and SOM activity and the effects of optogenetic perturbations. By reducing the model to a linear analytical form, we demonstrate that gamma oscillations emerge directly from the cell-specific connectivity of the three-population circuit. This connectivity motif alone accounts for experimentally observed phase relationships, with PV activity consistently leading that of SOM neurons. Together, this mean field model identifies a distinct structural mechanism giving rise to oscillations in canonical E–PV–SOM circuits and provides theoretical primitives for constructing large-scale, cell-type-specific models of cortical dynamics.

## Author summary

In this work, we develop a computational mean field model that predicts the collective electrical activity of populations of neurons composed of two main types of inhibitory cells, parvalbumin-positive (PV) and somatostatin-positive (SOM) neurons. We compare the predictions of this mean field model with simulations of large spiking neural networks composed of thousands of sparsely connected neurons. We show that this mean field model captures key features of the network, including how different inhibitory cell types shape rhythmic activity. In particular, we find that these inhibitory neurons play distinct roles in generating and controlling oscillatory rhythms at multiple frequencies. Using a linear approximation of the model, we show analytically that oscillations emerge from the specific connectivity scheme between these inhibitory cell types, rather than requiring precise tuning of signal delays or time constants. Because of its simplicity, this mean field model can be studied analytically and used as a building block for constructing large-scale brain models with area-specific interneuron composition and structured layers of inhibitory neurons.

## Introduction

Oscillations are a hallmark feature of neural dynamics, measurable from voltage membrane potentials to macroscopic electromagnetic waves, and are thought to play important roles in neural coding and signal transmission [1–7]. Their prevalence in electrophysiological recordings is mirrored by their natural emergence in computational models of interacting excitatory and inhibitory spiking neurons, as well as in purely inhibitory spiking neuronal networks [8–13]. However, while the theoretical underpinnings of oscillatory mechanisms in two-population models of excitatory and inhibitory neurons are well understood, we lack a theoretical understanding of cortical oscillations in more biologically realistic and heterogeneous microcircuits.

Mean-field models offer a powerful theoretical tool for dissecting the origin and stability of oscillations in neuronal networks. The pioneering Wilson–Cowan model showed that the population activity of Excitatory/Inhibitory (E/I) networks can be effectively captured by simple population equations, revealing the basic mechanisms underlying gamma oscillations [14]. Thanks to its simplicity and generality, it has become a standard model for describing canonical mechanisms driving oscillatory regimes in E/I networks such as Pyramidal–Interneuron Gamma (PING) and Interneuron Gamma (ING) in purely inhibitory networks. Consistent with Wilson-Cowan models, inhibitory and excitatory neurons show distinct phase relationships to gamma oscillations in several cortical and hippocampal circuits (e.g., [15–23]). Despite its elegance, the Wilson and Cowan model is a phenomenological model, as it is not derived directly from the spiking neural network it is supposed to represent. Over the years, considerable effort has been devoted to developing mean-field models capable of accurately reproducing the actual dynamics of spiking neural networks. In the context of quadratic integrate-and-fire (QIF) models, exact reductions have been derived to obtain low-dimensional representations of population dynamics [24]. However, these models miss key biological features of neural networks that strongly affect population dynamics, such as sparse connectivity between neurons [25] and employ *ad hoc* Cauchy distribution of heterogeneities or noise sources [26]. Other mean-field approaches have instead aimed to increase biophysical realism, for instance by incorporating conductance-based synapses and spike-frequency adaptation in sparse spiking neural networks that mimic *in vivo* circuits [27]. Regardless of the specific formulation, most of these models still rely on classical two-population E/I architectures, and thus on the PING or ING mechanisms proposed initially by the Wilson–Cowan framework.

Crucially, inhibitory neurons are not a homogeneous population but comprise distinct subtypes with specific circuit functions [28–32]. Among them, parvalbumin-expressing (PV) and somatostatin-expressing (SOM) interneurons have been shown to play complementary roles in controlling cortical oscillations [19,33,34]. Strikingly, during gamma oscillations, the experimentally observed phase delay occurs relatively high in SOM cell spikes, whereas PV interneurons tend to oscillate nearly in phase with the excitatory population [19]. These experimental findings challenge the traditional view of gamma and beta oscillations as emerging from a single, generic E–I mechanism, thus calling for a revised theoretical framework that explicitly accounts for interneuron diversity. Several recent studies have employed neural network models of E, PV, and SOM cells to investigate the role of distinct interneuron subtypes in gamma oscillations [35–38]. Other works have instead used phenomenological neural mass models to show that distinct interneuron types exert different effects on population dynamics [39–41]. In a recent work, we introduced the CAMINOS (Canonical Microcircuit Network Oscillations) model [42], a spiking neural network calibrated on experimental data, which proposed distinct roles of PV and SOM cells in generating and stabilizing gamma oscillations. While PV cells control mainly the frequency of oscillations, SOM cells control their amplitude. The CAMINOS model also was able to reproduce a broad range of experimental observations, in particular the effects of causal optogenetic manipulations targeting distinct interneuron classes during gamma oscillations. Numerical simulations revealed that a critical factor in the CAMINOS model was the specific asymmetric connectivity in canonical E-PV-SOM circuits, characterized by SOM cells exhibiting little to no self-inhibition and PV cells providing weak or no inhibition onto SOM cells.

However, because spiking network models are inherently high-dimensional and can only be explored through numerical simulations, it remains unclear whether the underlying computational mechanism in CAMINOS is distinct from a classical or effective PING model. Here, we take a crucial step by deriving a mean-field model of E–PV–SOM networks that preserves biological constraints on cellular properties and structural connectivity while remaining analytically tractable. We compared this mean-field model to direct numerical simulations of the corresponding spiking neural network and performed in silico manipulations on different interneuron populations within the mean-field framework - mimicking optogentic manipulations - to assess its capacity to predict experimental observations. We started with the biologically realistic mean-field model and progressively simplifyied the model to a linear skeleton, to analytically elucidate the existence of an oscillatory mechanism distinct from the classical PING model. Our results confirm that CAMINOS is a distinct oscillatory state emerging in three populations E-PV-SOM networks because of its special cell-specific connectivity structure.

## Results

The Results section is divided into two main parts. In the first part, we develop and analyze a biologically realistic mean-field model of the E–PV–SOM spiking network. We will investigate how this mean field model reproduces key experimentally observed features of cortical population dynamics. We will compare mean field prediction with direct numerical simulations of the corresponding neural network. In the second part, we construct a linear mean-field model that preserves the essential circuit architecture of the biologically realistic model, but allows for an exact analytical treatment.

### Biologically realistic mean-field model of pyramidal, PV, and SOM populations

**Fig 1:**
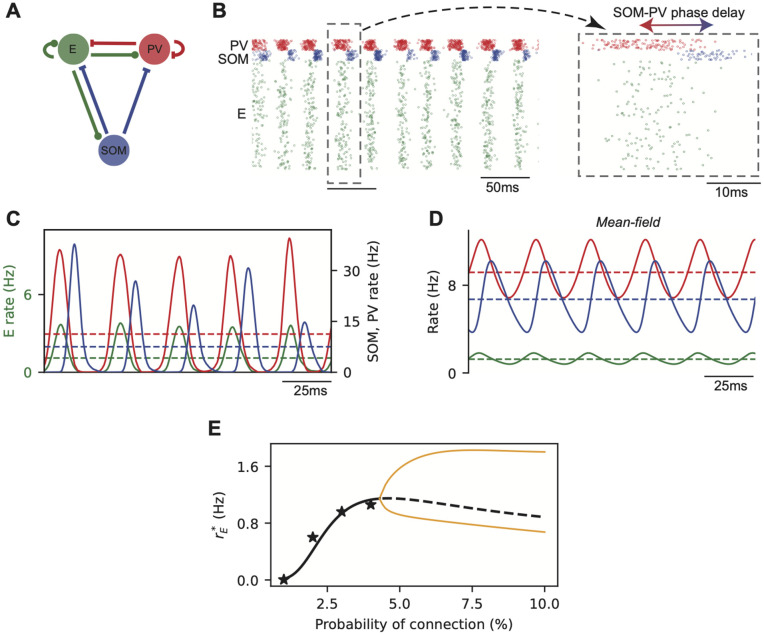
Biologically realistic mean field model setup and emergent dynamics. **(A)** Schematic of the E–PV–SOM circuit. **(B)** Raster plot of the simulated spiking network, with a zoomed-in view of one gamma cycle. **(C)** Population activity traces of the spiking network. **(D)** Corresponding mean-field simulation. In all panels, green represents E neurons, red represents PV interneurons, and blue represents SOM interneurons. **(E)** Stationary firing rate $r_{E}^{*}$ of E neurons in function of the probability of connection *p* (notice that *p* = 7% in panels B-D). Black continuous (dashed) line indicates stable (unstable) fixed point, orange lines indicates maximum and minimum value of *r*_*E*_ in time under a stable limit cycle. Stars are mean firing rates obtained from spiking neural network simulations.

We constructed a spiking network model, consisting of three cell types: excitatory pyramidal cells (E cells), PV interneurons, and SOM interneurons. The network comprises 8,000 excitatory cells and 1,500 inhibitory interneurons, with half of the interneurons being PV and the other half being SOM. We considered the network to be random and sparse, with each neuron forming a synapse on another neuron with a probability of 0.07. We designed the connectivity structure between cell populations based on [43], and we point out two key features: the absence of PV-to-SOM inhibition and the absence of self-inhibition of SOM. The dynamics of each neuron were modeled using the Adaptive exponential Integrate-and-fire (AdEx) model [44], and the couplings between neurons were modeled as conductance-based synapses. A synaptic delay of 2 ms was included for all synapses. We refer the reader to the Methods section, where detailed explanations, equations, and parameter values are provided. The schematic of the designed canonical microcircuit is shown in Fig 1A.

We have calibrated model parameters within biophysical ranges (see method section for details) and, for sufficiently large connectivity, the network generates oscillations, as can be observed from the raster plot shown in Fig 1B. We will show in the rest of the study how region-specific parameters such as the density of distinct cell types can affect the frequency of oscillations. Here, we fixed the density ratio of cell types from visual cortex as we directly compare with these data. Future works may employ this model to compare with different datasets from different brain regions and animal models. In this parameter setup, the emergent dynamics stabilizes in an oscillatory regime with an oscillation frequency of approximately 35 *Hz* within the low-gamma range, consistent with experimental results in the visual cortex during visual stimulation [19,33]. Fig 1B illustrates that SOM cells consistently fire with some delay relative to PV cells. This phase delay was estimated quantitatively across gamma cycles using the approach described in [42] and is found to be around 8.5 ms. The population activities of the spiking network are shown in Fig 1C, which illustrates that PV cells exhibit the highest mean firing rate, followed by SOM interneurons, and finally E cells with the lowest mean firing rate. We note that the mean firing rates (1–3 Hz for E and higher rates for SOM and PV), oscillation frequency, and phase delay between PV and SOM interneurons agree with previous experimental findings in the visual cortex of rodents during visual stimulation [19,33,45].

The next step was to derive a mean-field approximation and compare it to the spiking network. We used the mean field modeling approach developed for AdEx neurons in [27]. This approach basically employs a master equation formalism first developed in [46], that assumes the network in an asynchronous irregular state, memoryless over a time scale *T*. At the first order, the equations are formally the same as in the Wilson–Cowan model, but instead of a sigmoid, neuron’s firing activity is driven by biophysically grounded transfer functions. These transfer functions are derived from fluctuation-driven single-neuron dynamics, following the approach of [47] that we briefly summerise hereafter. In vivo–like cortical neurons, as neurons in our spiking network, operate in a high-conductance state [48] characterized by large synaptic conductances. This regime arises from dense, irregular Poissonian-like synaptic bombardment, which produces stochastic fluctuations in the membrane potential around a depolarized mean near a spiking threshold. From these fluctuations, one can construct a transfer function that deterministically maps the statistical properties of the membrane voltage—its mean ($\mu_{v}$), variance ($\sigma_{v}^{2}$), and effective time constant ($\tau_{v}$)—to the expected firing rate of a neuron. By expressing these membrane voltage statistics as functions of pre-synaptic population rates, the transfer function provides a principled input–output mapping for the population dynamics. One should note that generally $\mu_{v}$, $\sigma_{v}^{2}$, and $\tau_{v}$ are not constants and evolve through time. We refer the reader to the Methods section, where we provide a detailed explanation of the derivation of transfer functions. As we considered AdEx neurons we also introduced an adaptation variable *w* for each neuronal population in our mean-field equations, representing the average adaptation current within each group, see [27]. The two key parameters, *a* (subthreshold adaptation) and *b* (spike-triggered adaptation), control the contribution of membrane voltage fluctuations and spike events to this current, respectively. The adaptation time scale, denoted by $\tau_{w}$, is assumed to be much slower than the mean-field time scale *T* and therefore, on the mean-field timescale, the adaptation variable *w* can be treated as stationary, allowing the population firing rates to be computed conditionally on the current value of *w* while the slow adaptation dynamics evolve over a longer timescale. The firing rates determine the evolution of adaptation, while adaptation in turn modifies the membrane voltage statistics ($\mu_{v}$, $\sigma_{v}^{2}$, and $\tau_{v}$) and consequently affects the transfer function. In this way, the mean-field equations capture both fast fluctuation-driven dynamics and slow negative feedback from adaptation. The timescale of adaptation is the same as that of the spiking network (500 ms; see Methods section). The time constant *T* of the mean-field model is of the same order of the membrane time constant, and it was here chosen to be 15 ms such that the resulting dynamics reproduced the network’s temporal behavior and oscillation frequency. Finally, synaptic delay is introduced following the approach developed in [49].

The mean-field model is then described by the following set of Eq. 1 in which $\nu^{*}$ represents the population firing rates of the cell type *∈{E,PV,SOM}. ${ℱ}_{*}$ shows the transfer functions, $\nu_{t-\tau_{d}}^{*}$ denotes $\nu^{*}$ delayed by $\tau_{d}=2\;ms$ representing the synaptic delay. $E_{l}^{*}$ represents the resting membrane potential and $a_{*}$ and $b_{*}$ are the adaptation parameters with the same values as for the spiking network. It should be noted that the parameters of the spiking network, such as connection probabilities, number of neurons, synaptic properties, and neurons’ biophysical parameters, were incorporated in the derivation of the transfer functions. In other words, the transfer functions were calculated and fitted for specific values of the parameters of the spiking network. We refer the reader to the Methods section, where we provide a detailed explanation of the derivation of transfer functions based on the spiking network.


$$\begin{array}{cc} T\frac{d\nu^{E}}{dt} & =-\nu^{E}+{ℱ}_{E}\left(\nu_{t-\tau_{d}}^{E},\nu_{t-\tau_{d}}^{PV},\nu_{t-\tau_{d}}^{SOM},w^{E}\right)\\ T\frac{d\nu^{PV}}{dt} & =-\nu^{PV}+{ℱ}_{PV}\left(\nu_{t-\tau_{d}}^{E},\nu_{t-\tau_{d}}^{PV},\nu_{t-\tau_{d}}^{SOM}\right)\\ T\frac{d\nu^{SOM}}{dt} & =-\nu^{SOM}+{ℱ}_{SOM}\left(\nu_{t-\tau_{d}}^{E},\nu_{t-\tau_{d}}^{PV},\nu_{t-\tau_{d}}^{SOM},w^{SOM}\right)\\ \frac{dw^{E}}{dt} & =-\frac{w^{E}}{\tau_{w}}+b_{E}\nu^{E}+a_{E}\left(\mu_{v}^{E}\left(\nu_{t-\tau_{d}}^{E},\nu_{t-\tau_{d}}^{PV},\nu_{t-\tau_{d}}^{SOM},w^{E}\right)-E_{l}^{E}\right)\\ \frac{dw^{SOM}}{dt} & =-\frac{w^{SOM}}{\tau_{w}}+b_{SOM}\nu^{SOM}+a_{SOM}\left(\mu_{v}^{SOM}\left(\nu_{t-\tau_{d}}^{E},\nu_{t-\tau_{d}}^{PV},\nu_{t-\tau_{d}}^{SOM},w^{SOM}\right)-E_{l}^{SOM}\right) \end{array}$$ (1)

Fig 1D shows the mean-field simulation for the same parameters employed for network simulations in Fig 1B and 1C, which produces a robust oscillatory regime at 35 *Hz*, with a PV–SOM phase delay of approximately 7 *ms*. We observe a good agreement between the mean-field and spiking network models in key proprieties, such as mean firing rates, frequency of oscillation, and PV–SOM phase delay. It is important to notice that the peak of activity of SOM and PV is higher in the network simulations with respect to the mean field model. It is natural to expect some quantitative discrepancies in this model, given the approximations performed in the transfer function evaluation. Nevertheless, the agreement with a sparse biophysical networks is overall very good in terms of dynamical behavior and key quantities in relation to experimental data (mean rates, oscillation frequency and neurons’ spike timing). We have also performed a stability analysis of the mean field model, showing that oscillations are lost for highly sparse networks. Indeed, oscillations emerge for sufficiently connected networks when the stable fixed point becomes unstable (see Fig 1E) in function of the overall probability of connection *p*. Notice that we observe a good agreement between network simulations and mean field predictions of the stationary firing rates in asynchronous regimes (see stars in Fig 1E)

Throughout the study, we define the mean-field (and the corresponding spiking network) described in this section as the “reference model”. In subsequent sections, when we refer to the reference model, we mean this original setup.

### Distinct contributions of PV and SOM interneurons to gamma oscillations

In experiments, optogenetic silencing has been used to investigate the causal contributions of specific interneuron classes to cortical dynamics [33,34]. We dissected the contributions of PV and SOM interneurons in our oscillation-generating model using two types of analyses, which we term “silencing” and “randomizing.” By silencing a given proportion of a cell type, we mean reducing the number of neurons of that type by the same proportion in the reference model. By randomizing a given proportion of a cell type, we mean randomizing the spike times of that proportion that we achieve by replacing that fraction of neurons with Poisson units emitting spikes at a constant rate equal to the mean population activity of that cell type in the reference model. Affecting overall inhibition, silencing effects are compatible with a purely modulatory influence of one population over others. This is similar to changes in neuronal excitability, whose effects could be investigated in future studies as a point of comparison. Randomization, on the other hand, while maintaining the same net inhibition, tests the causal role of synchronization within a specific cell population in the emergence of oscillations.

**Fig 2:**
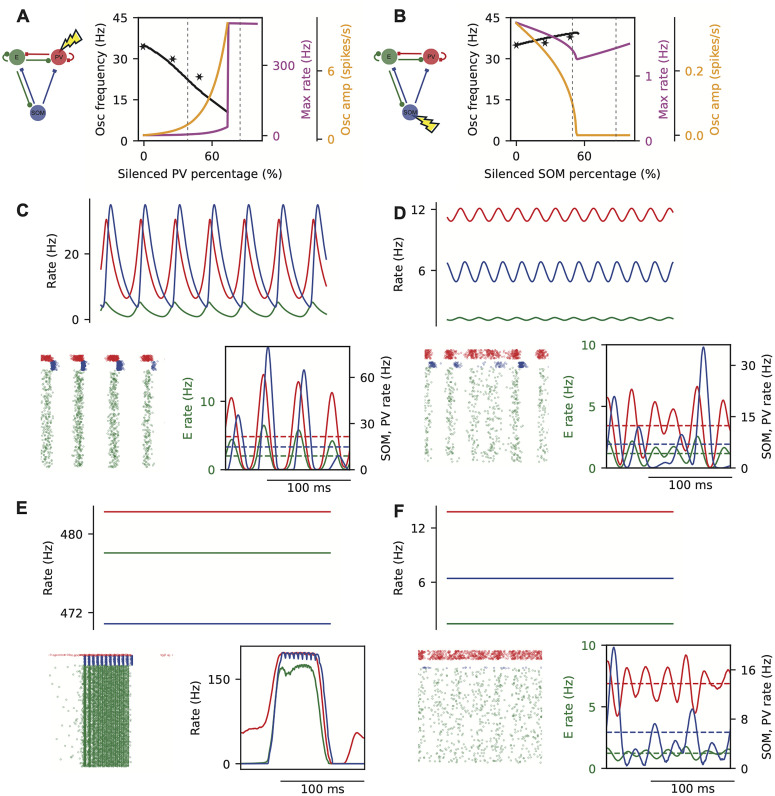
Emergent dynamics across different levels of interneuron silencing. Panels A-C-E shows the results of silencing PV interneurons, and B-D-F shows the corresponding results for silencing SOM interneurons. Panel (A) depicts the mean-field characteristics across different levels of PV silencing: the black line indicates oscillation frequency (stars are obtained from numerical simulations of the spiking neural network), the orange line indicates oscillation amplitude, and the magenta line shows the maximum firing rate of the excitatory population. Panels C and D present the mean-field activity (top), spiking network raster plot and corresponding firing activity (bottom) when 40% of PV interneurons were silenced (see vertical dashed lines in panel A and B). Panels E and F show the same as C and D when 90% of PV interneurons were silenced. In all panels, green represents excitatory neurons, red represents PV interneurons, and blue represents SOM interneurons.

Fig 2A shows the mean-field dynamics for PV silencing levels ranging from 0% to 100%. Increasing the proportion of silenced PV neurons results in elevated firing rates and larger oscillation amplitudes, accompanied by a reduction in oscillation frequency. Notice the good quantitative agreement between mean field model and network simulations in terms of oscillations frequency (see stars in panel A that have been obtained from network simulations). We can appreciate the comparison between network simulations and mean field model in the traces of E, PV and SOM population activity reported in Fig 2C. We observe a good quantitative agreement in terms of the average firing rate, oscillations’ frequency but some discrepancies on the amplitude of oscillations (in particular for interneurons). This may be due to the fitting procedure of the transfer function and to finite size effects. Indeed, we observe that network simulations show less regular oscillations with respect to mean field model, pointing to finite size endogenous noise. When the fraction of silenced PV neurons exceeds 70%, the system undergoes a phase transition into a seizure-like regime characterized by the maximum firing rate of the excitatory population, equal to the inverse of the refractory period. Notice that our mean field approach cannot predict any hyper fast synchronisation, due to the Markovian approximation over the time scale T~15ms. Such hypersynchronisation, that we observe in network simulations for strong PV silencing, is represented in the mean field model by a stable fixed point at an aberrant firing rate of the order of the refractory period (see Fig 2E). We consider such fixed point with aberrant high rate a signature of an hypersynchronised epileptic-like state in the corresponding network simulations.

**Fig 3:**
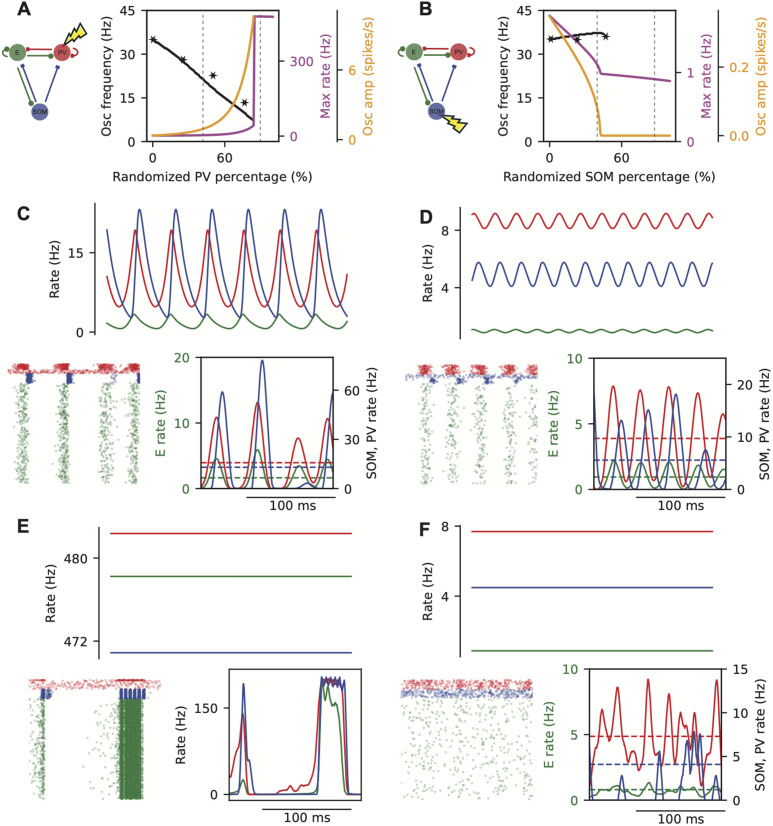
Emergent dynamics across different levels of interneuron spike times’ randomization. Panels A-C-E shows the results for randomizing PV interneuron spike times, and B-D-F shows the corresponding results for SOM interneurons. Panel (A) depicts the mean-field dynamics across different levels of PV spike-time randomization: the black line indicates oscillation frequency (stars are obtained from numerical simulations of the spiking neural network), the orange line indicates oscillation amplitude, and the magenta line shows the maximum firing rate of the excitatory population. Panels C and D present the mean-field activity (top), spiking network raster plot and corresponding firing activity (bottom) when 40% of PV interneurons were randomized (see vertical dashed lines in panel A and B). Panels E and F show the same as C and D when 90% of PV interneurons were randomized. In all panels, green represents excitatory neurons, red represents PV interneurons, and blue represents SOM interneurons.

Randomization of PV neurons produced qualitatively similar outcomes, with minor variations (Fig 3A). Results from network simulations and mean-field traces for 40% and 90% PV randomization are also shown in Fig 3C and 3E. Also for randomization, we observe a similar agreement between network simulations and mean field model as for the case of silencing: good mean rates, oscillations’ frequencies but some discrepancies in oscillation’s amplitude for interneurons (see network simulations vs mean field model traces). Overall, mean field predictions are consistent with network simulations and with experimental observations [33].

Panels B,D and F in Fig 2 show the mean-field and network dynamics under different levels of SOM silencing. Increasing the proportion of silenced SOM neurons leads to a progressive reduction in oscillation amplitude, and at 60% silencing, the system shows a transition into a stable non-oscillatory regime. Compared with the control condition shown in Fig 1C (and Fig 1D for the mean-field model), oscillation amplitudes in the network progressively decreased as the level of silencing increased. This effect is illustrated by the excitatory population traces in Fig 2D and 2F, corresponding to 40% and 90% silencing, respectively. Notice that the amplitude of oscillations does not drop to zero in the network simulations (at variance with respect to the mean field model, see the orange trace in panel B) due to finite size fluctuations. Also, the amplitude of oscillations of PV and SOM cells is larger in network simulations with respect to the mean field model predictions (this discrepancy was observed also for PV silencing). We observe a good agreement between the mean activity of cell types and network simulations (see dashed lines for network simulations in bottom panels of Fig 2C and 2D). Moreover, the oscillation frequency also increases slightly with SOM silencing, in good quantitative agreement between mean field and network simulations (see stars in panel B), although this effect was much smaller than the frequency shift observed during PV silencing. These findings recapitulate previous experimental observations [33,34] and show a good agreement between mean field model and network simulations of optogenetic manipulations, apart from some discrepancies in interneurons amplitude of oscillations between mean field and network model.

Randomization of SOM neurons produced qualitatively similar outcomes, with minor variations (Fig 3B). Representative raster plots, traces from network simualtions and mean-field traces for 40% and 90% SOM randomization are also shown in Fig 3D and 3F. Overall, these results, consistent with experimental findings, demonstrate that SOM interneurons, and their spike timing, play a key role in sustaining oscillatory amplitude, as their silencing and or randomizing abolishes rhythmic activity.

### Gradients in interneuron densities shape oscillatory regimes and vulnerability to epilepsy

**Fig 4:**
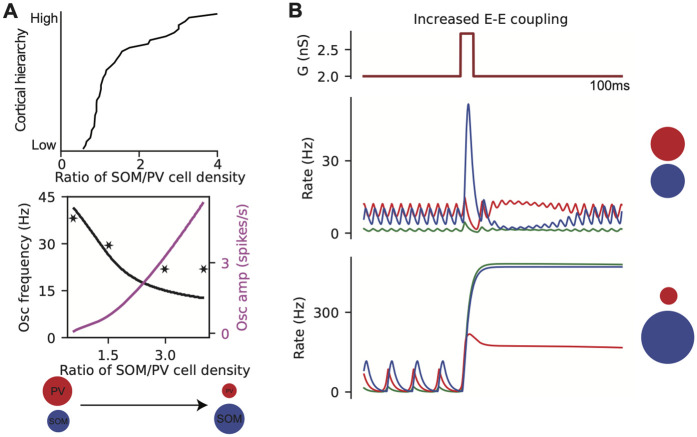
Influence of SOM/PV density ratio gradients on the emergent mean field dynamics. **(A)** Top panel: density ratio of SOM to PV interneurons across the cortical hierarchy, extracted from [52]. (A) Bottom panel: meanfield dynamics at different SOM/PV density ratios, with the black line indicating oscillation frequency and the magenta line indicating oscillation amplitude (stars are obtained from numerical simulations of the spiking neural network). **(B)** Effect of a transient increase in excitatory self-coupling. The top panel of column (B) shows the applied increase transient in excitatory self-coupling. The middle panel presents the mean-field response to this transient when *SOM*/*PV* = 1, and the bottom panel shows the corresponding response when *SOM*/*PV* = 4.

PV and SOM interneurons exhibit distinct laminar and regional distributions. PV cells reach their peak density in cortical layer 4, whereas SOM interneurons are most abundant in layer 5 [50,51]. Moreover, the density ratio of SOM to PV interneurons varies systematically across cortical regions, forming a gradient from early sensory areas to higher-order association cortices as shown in the top panel of Fig 4A [52]. In primary sensory regions, the SOM/PV ratio is relatively low, indicating a predominance of PV interneurons, whereas in higher-order regions such as the PFC, the ratio is much higher, reflecting the great prevalence of SOM cells. Our model directly links oscillation frequency to the density of SOM and PV interneurons across cortical layers and regions. When the SOM/PV density ratio is high, the model generates slower oscillations. Conversely, when the SOM/PV density ratio is low, as in upper layers and/or sensory/motor cortices, the model produces faster oscillations. This correspondence demonstrates that variations in interneuron composition can account for the laminar and regional patterns of oscillatory activity observed experimentally. The mean-field predictions (and the corresponding network simulations) under different SOM/PV density ratios are shown in Fig 4A (bottom panel). As illustrated, increasing the SOM/PV ratio decreases the oscillation frequency while increasing its amplitude. This result is consistent between network simulations and mean field model (see stars in Fig 4A), even if the decrease in frequency is stronger in the mean field model for large SOM/PV ratio. Notably, when varying the SOM/PV density ratio, the total number of interneurons was kept fixed at 1500, with only the relative numbers of SOM and PV cells adjusted.

Next, we asked whether the balance between SOM and PV interneurons influences the stability of dynamics. To test this, we introduced a transient increase in excitatory-to-excitatory (E–E) coupling. As shown in Fig 4B, the setup with a high SOM/PV ratio was more susceptible to instability under this perturbation, exhibiting seizure-like dynamics marked by a phase transition. In contrast, network configurations with a low SOM/PV ratio remained stable when the recurrent excitation transiently increased, both in network simulations and mean field models. This is different from the results obtained in [42], where SOM/PV ratio lower than 1 already led to instability under local perturbations. This discrepancy likely arises from differences in parameter settings; notably, the present study incorporates synaptic delays, which were absent in [42]. Future work could further investigate how such delays influence the local stability of E–PV–SOM networks.

### Connectivity architecture, not synaptic kinetics or adaptation, as the fundamental mechanism of oscillation generation

Firstly, we investigate how the specific connectivity patterns contribute to the emergence of oscillatory activity and ask whether the particular arrangement of connections between cell types underlies the generation of oscillatory regimes. The key aspect of the reference circuit is that the SOM population neither inhibits itself nor receives inhibition from the PV population. To assess the functional relevance of this wiring scheme, we systematically introduce these missing connections one at a time and examine their individual impact on network dynamics.

**Fig 5:**
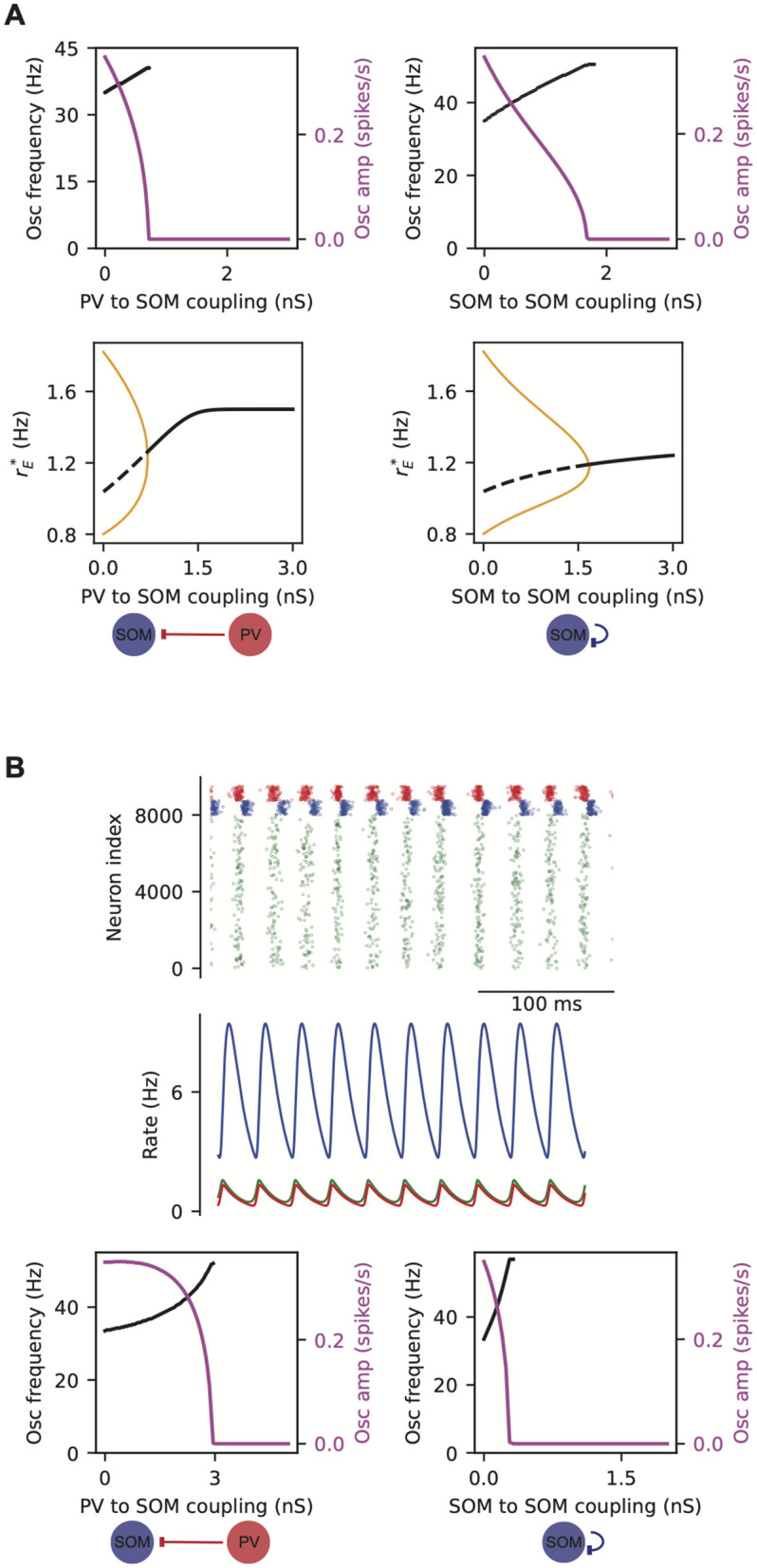
Role of SOM to SOM and PV to SOM connections (A) Left: effect of PV → SOM inhibition on the mean-field dynamics. Right: effect of SOM self-inhibition on the mean-field dynamics. Top panels show oscillations frequency and amplitude, while lower panels show the bifurcation diagram obtained from stability analyses: black continuous (dashed) line indicates stable (unstable) fixed point and orange indicates stable limit cycles (maximum and minimum values). **(B)** Results for the reduced system (without synaptic delay and adaptation). Top: raster plot from spiking neural network simulations and corresponding mean-field dynamics. Bottom: effects of PV → SOM inhibition (left) and SOM self-inhibition (right) on the reduced mean-field dynamics.

Fig 5A illustrates the effect of introducing additional inhibitory connections into the reference model. When a coupling from PV to SOM is added, increasing the strength of this coupling progressively reduces the amplitude of oscillation until a critical point is reached, at which a phase transition occurs and the system shifts into a non-oscillatory, stable regime. A similar effect is observed when self-inhibition is introduced onto the SOM population: strengthening this coupling also diminishes oscillation amplitude and eventually drives the system into a non-oscillatory stable state. However, the impact of the PV → SOM connection is much more pronounced, as the transition to the non-oscillatory regime occurs at lower connectivity strengths compared to the case of SOM self-inhibition. It should be noted that these additional inhibitory couplings (PV → SOM and SOM → SOM) were implemented within the same network framework described previously. As with the other connections, they were introduced as sparse, random synapses with the same connection probability (7%) and modeled using conductance-based synapses with identical synaptic delays of 2 *ms*.

We next asked whether the fundamental mechanism underlying oscillatory dynamics lies solely in the connectivity architecture of the circuit, with other factors such as synaptic time constants/delay and spike frequency adaptation serving only as modulators rather than essential components. To test this hypothesis, we removed both synaptic timescales and adaptation from the model. In this simplified configuration, we also set the SOM leak reversal potential to $E_{L}^{SOM}=-75\text{ mV}$ (to prevent the SOM overactivity) and provided external input only to the excitatory population, with a rate of 1 Hz. After removing synaptic timescales and adaptation, the mean-field equations take the following form:


$$\begin{array}{cc} T\frac{d\nu^{E}}{dt} & =-\nu^{E}+{ℱ}_{E}\left(\nu^{E},\nu^{PV},\nu^{SOM}\right)\\ T\frac{d\nu^{PV}}{dt} & =-\nu^{PV}+{ℱ}_{PV}\left(\nu^{E},\nu^{PV},\nu^{SOM}\right)\\ T\frac{d\nu^{SOM}}{dt} & =-\nu^{SOM}+{ℱ}_{SOM}\left(\nu^{E},\nu^{PV},\nu^{SOM}\right) \end{array}$$ (2)

The mean-field Eq. 2 represent the version of the reference model without synaptic timescales/delay and adaptation. The upper panel of Fig 5B illustrates the simulation of 2. In particular, even in the absence of synaptic and adaptation timescales, the system continues to generate oscillations in the low gamma range (the oscillation frequency is around 35 Hz). Moreover, PV activity consistently leads SOM activity in phase. These results demonstrate that the core mechanism driving this type of oscillation does not rely on synaptic timescales or the biophysical properties of neurons, such as adaptation. Instead, it suggests that the essential factor is the specific connectivity architecture—namely, the minimal or absent PV → SOM inhibition and the weak or absent SOM self-inhibition.

To further verify our findings, we reintroduced the PV → SOM and SOM → SOM connections separately into the reduced mean-field 2 and examined their effects on the dynamics. The lower left panel of Fig 5B shows the results for the PV → SOM case: as the strength of this connection increases, the amplitude of oscillations progressively declines until a phase transition occurs, driving the system into a non-oscillatory stable regime. Similarly, the lower right panel of Fig 5B illustrates the effects of adding self-inhibition to the SOM. Strengthening this connection likewise reduces the oscillation amplitude, and beyond a critical point, the system again transitions into a non-oscillatory stable state.

In summary, these analyses demonstrate that the essential driver of the oscillatory regime is the specific connectivity architecture, characterized by little to no PV → SOM inhibition and minimal SOM self-inhibition, rather than synaptic or adaptation timescales. This emphasizes that the structural wiring of the network, rather than its biophysical details, forms the fundamental basis of the oscillatory dynamics.

### Linear mean-field model

Assuming that a general equilibrium exists in the state space of a biological circuit composed of three cell types, one can reasonably expect that the system behaves linearly in the vicinity of this equilibrium. A linear approximation is then often valid for describing the dynamics around the equilibrium. On this basis, we develop and analyze a linear model of the E-PV-SOM circuit, demonstrating that some important features of the original system are retained in this simplified linear framework. This part presents a fully analytical development of the linear model; however, to keep the exposition accessible to a wide readership, we include the detailed mathematical proofs and technical arguments in the Appendixes of S1 Text.

### Framework for the general linear rate model

We model the dynamics of population activity in a local cortical microcircuit comprising excitatory (E) neurons, parvalbumin-expressing (PV) interneurons, and somatostatin-expressing (SOM) interneurons. The population activity vector is defined as


$$\begin{array}{c} \nu (t)=[\begin{array}{c} \nu_{E}(t)\\ \nu_{PV}(t)\\ \nu_{SOM}(t) \end{array}], \end{array}$$

where each component represents the average firing rate of the corresponding neuronal population at time *t*. The temporal evolution of these activities is described by the linear rate model:


$$\begin{array}{c} \tau \frac{d\nu (t)}{dt}=-\nu (t)+Q\nu (t), \end{array}$$ (3)

where τ denotes the time constant of population activity, and *Q* is the effective connectivity matrix:


$$\begin{array}{c} Q=[\begin{array}{ccc} Q_{E\to E} & -Q_{PV\to E} & -Q_{SOM\to E}\\ Q_{E\to PV} & -Q_{PV\to PV} & -Q_{SOM\to PV}\\ Q_{E\to SOM} & 0 & 0 \end{array}], \end{array}$$

with $Q_{*}>0$ representing the effective coupling strength between populations. Positive entries correspond to excitatory projections originating from E neurons, while negative entries represent inhibitory influences mediated by PV and SOM interneurons. In this abstraction, we omit the inhibitory connections PV→SOM and SOM→SOM, consistent with experimental findings indicating that such synapses are very sparse or functionally negligible in cortical networks.

The term −ν(t) in Eq. 3 describes the intrinsic relaxation of population activity toward baseline in the absence of input. Biologically, this captures processes such as membrane leakage or adaptation that cause neural activity to decay when it is not sustained by excitation.

Defining A=Q−I, where *I* denotes the identity matrix, the system can be rewritten in the standard linear form:


$$\begin{array}{c} \frac{d\nu (t)}{dt}=\frac{1}{\tau }A\nu (t), \end{array}$$ (4)

with


$$\begin{array}{c} A=[\begin{array}{ccc} Q_{E\to E}-1 & -Q_{PV\to E} & -Q_{SOM\to E}\\ Q_{E\to PV} & -Q_{PV\to PV}-1 & -Q_{SOM\to PV}\\ Q_{E\to SOM} & 0 & -1 \end{array}]. \end{array}$$

This formulation highlights the interplay between recurrent excitation and inhibition in shaping the temporal evolution of population activity. In general, the solution to Eq. 4 can be expressed through the eigen-decomposition of *A*:


$$\begin{array}{c} \nu (t)=c_{1}e^{\lambda_{1}t}\nu_{1}+c_{2}e^{\lambda_{2}t}\nu_{2}+c_{3}e^{\lambda_{3}t}\nu_{3}, \end{array}$$

where $\lambda_{1},\lambda_{2},\lambda_{3}$ are the eigenvalues of $\frac{1}{\tau }A$ and $\nu_{1},\nu_{2},\nu_{3}$ are the corresponding eigenvectors. The constants *c*_1_, *c*_2_, *c*_3_ are uniquely determined by the initial population activity ν(0).

Each eigenmode $\left(\lambda_{i},\nu_{i}\right)$ represents a distinct dynamical motif of the circuit, corresponding to coordinated patterns of population activity. The nature of the eigenvalues determines whether these modes decay, oscillate, or grow over time, thereby linking the circuit’s connectivity structure to its emergent temporal dynamics. We first clarify some terminology that we use regarding oscillatory dynamical regimes. Generally, oscillatory dynamics occur when the matrix *A* has a pair of complex conjugate eigenvalues and a negative (≤0) real eigenvalue. The damped oscillation arises when the complex conjugate pair has a strictly negative real part. The pure oscillation occurs when the complex conjugate pair is purely imaginary. The growing oscillation arises when the complex conjugate has a strictly positive real part.

### If E-PV-SOM circuit oscillates, then E and PV activity lead SOM activity in phase

In the linear system Eq. 4, oscillatory dynamics arises when the matrix *A* has complex-conjugate eigenvalues. Specifically, if *A* possesses an eigenvalue of the form α+iω with ω>0, the corresponding eigenmode produces oscillations at frequency ω/(2πτ).

One of the key dynamical features of gamma oscillations in the E-PV-SOM circuit, as noted earlier, is that PV interneurons activity leads SOM cells activity in phase. To represent this phenomenon within the linear system, we note that if


$$\begin{array}{c} \nu_{\alpha +i\omega }=[\begin{array}{c} \nu_{1}\\ \nu_{2}\\ \nu_{3} \end{array}] \end{array}$$

is an eigenvector of *A* associated with the eigenvalue α+iω (ω>0), then the corresponding oscillatory mode exhibits a well-defined phase relationship among its components. In particular, a phase lead of the PV population relative to the SOM population is expressed by


$$\begin{array}{c} \text{arg}(\nu_{2})>\text{arg}(\nu_{3}), \end{array}$$

or equivalently, if $\nu_{2}=r_{2}e^{i\theta_{2}}$ and $\nu_{3}=r_{3}e^{i\theta_{3}}$, then $\theta_{2}>\theta_{3}$.

In the following proposition, we prove that for any set of parameters $Q_{*}>0$, if *A* admits a non-real eigenvalue α+iω (ω>0) with corresponding eigenvector $\nu =[\nu_{1},\;\nu_{2},\;\nu_{3}{]}^{⊤}$, the phases of $\nu_{1}$ (E population) and $\nu_{2}$ (PV population) are strictly larger than that of $\nu_{3}$ (SOM population). In other words, whenever Eq. 3 exhibits oscillatory behavior, the E and PV populations necessarily lead the SOM population in phase. We present the proposition here; the full mathematical proof, including technical details, is given in Appendix A in S1 Text.

**Proposition 1.** *Suppose that the matrix*


$$\begin{array}{c} A=[\begin{array}{ccc} Q_{E\to E}-1 & -Q_{PV\to E} & -Q_{SOM\to E}\\ Q_{E\to PV} & -Q_{PV\to PV}-1 & -Q_{SOM\to PV}\\ Q_{E\to SOM} & 0 & -1 \end{array}] \end{array}$$

*in which all* $Q_{*}$ *are strictly positive, has a non-real complex eigenvalue* α+iω *and* $\nu =[\nu_{1},\nu_{2},\nu_{3}{]}^{T}$ *is the corresponding eigenvector. Then the phases of* $\nu_{1}$ *and* $\nu_{2}$ *are strictly larger than the phase of* $\nu_{3}$.

### Conditions for sustained oscillations in the E–PV–SOM linear system

In this section, we determine the conditions that must be maintained so that the solution of this system is purely oscillatory (not growing or damped oscillations). Having such a solution implies that $\frac{1}{\tau }A$ has a pair of pure imaginary ±iω,(ω>0) and a real *r* ≤ 0 eigenvalues. The characteristic polynomial of $\frac{1}{\tau }A$ is


$$\begin{array}{c} \left|\begin{array}{c} \frac{1}{\tau }[\begin{array}{ccc} Q_{E\to E}-1-\lambda \tau & -Q_{PV\to E} & -Q_{SOM\to E}\\ Q_{E\to PV} & -Q_{PV\to PV}-1-\lambda \tau & -Q_{SOM\to PV}\\ Q_{E\to SOM} & 0 & -1-\lambda \tau \end{array}] \end{array}\right| \end{array}$$

and must be equal to $\left(\lambda +r)(\lambda^{2}+\omega^{2}\right)$. In other words, $\det\left(\frac{1}{\tau }A-\lambda I\right)=(\lambda -r)(\lambda^{2}+\omega^{2})$ is required.

Expanding both sides and then matching the coefficients, yields the necessary and sufficient conditions as follows.


$$\begin{array}{c} r=Q_{E\to E}-Q_{PV\to PV}-3\leq 0 \end{array}$$ (5)

$$\begin{array}{c} \omega^{2}=\frac{1}{\tau^{2}}(-Q_{E\to E}Q_{PV\to PV}+Q_{E\to PV}Q_{PV\to E}-2Q_{E\to E}+2Q_{PV\to PV}+Q_{E\to SOM}Q_{SOM\to E}+3)>0 \end{array}$$ (6)

$$\begin{array}{cc} & Q_{SOM\to PV}=\frac{1}{Q_{E\to SOM}Q_{PV\to E}}(-Q_{E\to E}^{2}Q_{PV\to PV}-2Q_{E\to E}^{2}+Q_{PV\to PV}^{2}Q_{E\to E}+6Q_{E\to E}Q_{PV\to PV}\\ & \;\;\;+8Q_{E\to E}-2Q_{PV\to PV}^{2}-8Q_{PV\to PV}+Q_{PV\to E}Q_{E\to PV}(Q_{E\to E}-Q_{PV\to PV}-2)\\ & \;\;\;\;\;\;+Q_{SOM\to E}Q_{E\to SOM}(Q_{E\to E}-2)-8) \end{array}$$ (7)

These three conditions also give us some intuitive insights into the system. For example, from 5, we understand that self-excitation cannot be much larger than PV self-inhibition. Eq. 6, which is also an explicit expression of oscillation frequency ($f=\frac{\omega }{2\pi }$), indicates that the oscillation frequency is mainly determined by the time constant τ since it depends linearly on $\frac{1}{\tau }$ whereas it depends on the square root of $Q_{*}$. The oscillation frequency is also an increasing function of $Q_{SOM\to E}$ and $Q_{E\to SOM}$. Another property is that the oscillation frequency does not depend on $Q_{SOM\to PV}$.

### Parameter selection and dynamical regimes

The next stage is to assign values to parameters in order to specify our model. To this end, we first consider a system consisting solely of E and PV, assign values to its parameters to generate a damped oscillation, and demonstrate how adding SOM to this system sustains the oscillation. We consider that the system is $\frac{d\nu }{dt}=\frac{1}{\tau }B\nu$ where


$$\begin{array}{c} B=[\begin{array}{cc} Q_{E\to E}-1 & -Q_{PV\to E}\\ Q_{E\to PV} & -Q_{PV\to PV}-1 \end{array}]. \end{array}$$

To have a damped oscillation in the E-PV system, the eigenvalues of *B* must be $\gamma \pm i\omega^{\prime }$ such that $\gamma <0,\;\omega^{\prime }>0$. For these eigenvalues to exist, the following two conditions must be met.


$$\begin{array}{c} Tr(B)=Q_{E\;to\;E}-Q_{PV\;to\;PV}-2<0 \end{array}$$ (8)

$$\begin{array}{c} (Tr(B){)}^{2}-4\det(B)<0 \end{array}$$ (9)

Assigning values to $Q_{E\to E}$, $Q_{E\to PV}$, $Q_{PV\to E}$, and $Q_{PV\to PV}$ must be such that it satisfies 8 and 9. We take $Q_{E\to E}=5$, $Q_{E\to PV}=5$, $Q_{PV\to E}=5$ and $Q_{PV\to PV}=4$.

To incorporate SOM into the system, it is important to note that satisfying 8 and 9 automatically ensures that 5 and 6 are also satisfied. Therefore, any choice of values for $Q_{E\to SOM}$, $Q_{SOM\to E}$, and $Q_{SOM\to PV}$ will inherently meet 5 and 6. Consequently, when selecting $Q_{E\to SOM}$, $Q_{SOM\to E}$, and $Q_{SOM\to PV}$, only 7 must be imposed.

We choose $Q_{E\to E}=5$, $Q_{E\to PV}=5$, $Q_{PV\to E}=5$, $Q_{PV\to PV}=4$, $Q_{E\to SOM}=4$. Then 7 gives $Q_{SOM\to PV}=\frac{6}{10}Q_{SOM\to E}-\frac{7}{20}$. We choose $Q_{SOM\to E}=4$ and so $Q_{SOM\to PV}=2.05$. One should note that choosing these values for $Q_{*}$ is only an example. In addition, we take τ to be 20 *ms*. Therefore, our base linear model is:


$$\begin{array}{c} \frac{d}{dt}[\begin{array}{c} \nu_{E}(t)\\ \nu_{PV}(t)\\ \nu_{SOM}(t) \end{array}]=\frac{1}{0.02}[\begin{array}{ccc} 4 & -5 & -4\\ 5 & -5 & -2.05\\ 4 & 0 & -1 \end{array}][\begin{array}{c} \nu_{E}(t)\\ \nu_{PV}(t)\\ \nu_{SOM}(t) \end{array}] \end{array}$$ (10)

**Fig 6:**
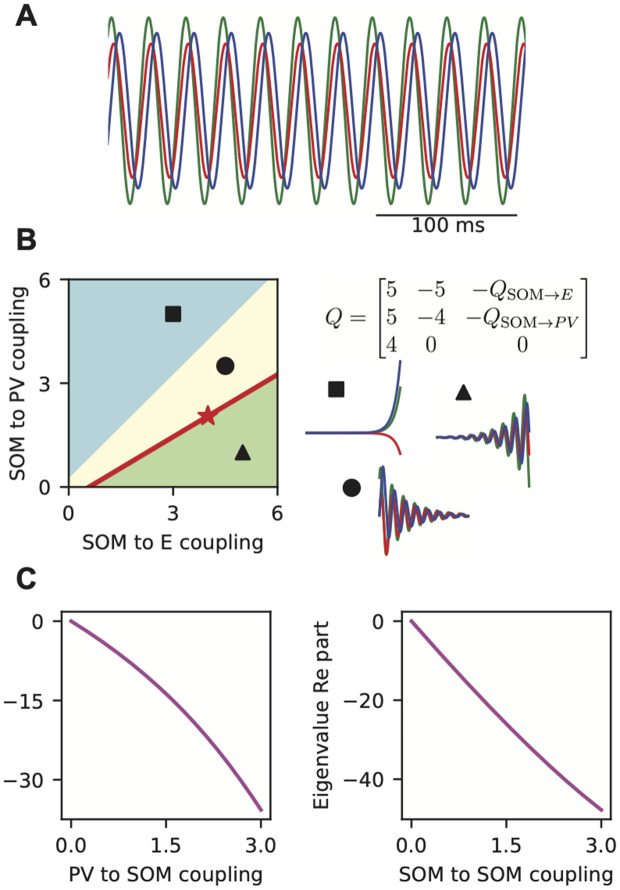
Linear mean-field analysis and phase diagram. **(A)** Linear mean-field dynamics at the parameter set indicated by the red star in (B). **(B)** Phase diagram of the linear mean-field system, with SOM → E strength on the x-axis and SOM → PV strength on the y-axis. Colors indicate different dynamical regimes: pale blue, coupling matrix with one positive real eigenvalue and a pair of complex conjugates with negative real parts; pale yellow, one negative real eigenvalue and a pair of complex conjugates with negative real parts; pale green, one negative real eigenvalue and a pair of complex conjugates with positive real parts. The red line marks the locus of purely imaginary eigenvalues, and the red star indicates the parameter point corresponding to (A). **(C)** Effect of adding additional inhibitory connections to the mean-field system at the red-star point. Left: PV → SOM inhibition. Right: SOM → SOM inhibition. In both cases, the system transitions from having a pair of purely imaginary eigenvalues to a pair of complex conjugates with negative real parts; moreover, increasing the strength of these added connections shifts the real part further into the negative range.

One can see the activity dynamics of the base linear model 10 in Fig 6A. The frequency of oscillation is around 40 *Hz*, and PV has a 4 *ms* phase advance relative to SOM.

Next, we consider the system such that all $Q_{*}$s are fixed to the base linear model 10 values except $Q_{SOM\to E}$ and $Q_{SOM\to PV}$, which are treated as free parameters, and to investigate the dynamical regimes of the system. In order to characterize the dynamical regimes, the eigenvalues of the matrix


$$\begin{array}{c} F=[\begin{array}{ccc} 4 & -5 & -Q_{SOM\to E}\\ 5 & -5 & -Q_{SOM\to PV}\\ 4 & 0 & -1 \end{array}] \end{array}$$

should be investigated. As shown in Appendix B in S1 Text, for any choice of $Q_{SOM\to E}$ and $Q_{SOM\to PV}$, the matrix *F* always possesses one real eigenvalue and a pair of complex conjugate eigenvalues.

Fig 6B illustrates the dynamical regimes of


$$\begin{array}{c} \frac{d}{dt}[\begin{array}{c} \nu_{E}(t)\\ \nu_{PV}(t)\\ \nu_{SOM}(t) \end{array}]=\frac{1}{0.02}[\begin{array}{ccc} 4 & -5 & -Q_{SOM\to E}\\ 5 & -5 & -Q_{SOM\to PV}\\ 4 & 0 & -1 \end{array}][\begin{array}{c} \nu_{E}(t)\\ \nu_{PV}(t)\\ \nu_{SOM}(t) \end{array}]. \end{array}$$ (11)

The red line, $Q_{SOM\to PV}=\frac{6}{10}Q_{SOM\to E}-\frac{7}{20}$, represents the dynamics where the solutions of the system are pure oscillatory. The point shown on the red line (by a red star) represents the setting mentioned above (where $Q_{SOM\to E}=4$ and $Q_{SOM\to PV}=2.05$). Below the red line (pale green), is the regime where the real part of the complex eigenvalues is positive and the real eigenvalue is negative and therefore the system is unstable (more precisely, the solutions are growing oscillations). Above the red line, the pale yellow region, is the regime where the real part of the complex eigenvalues is negative and the real eigenvalue is also negative. Therefore, the pale yellow regime is a stable regime or, more precisely, a damped oscillation. Pale blue is where the real part of complex eigenvalues is negative but the real eigenvalue is positive and consequently, the system is unstable. The boundary between pale blue and pale yellow is the line *h* = 0 or more precisely $Q_{SOM\to PV}=Q_{SOM\to E}+0.25$ where the real eigenvalue is 0.

### Effects of PV→SOM and SOM→SOM couplings on the linear model

Here, we consider the base linear model 10 and firstly wonder what happens if we add a PV → SOM coupling to it. Hence, we need to investigate the system:


$$\begin{array}{c} \frac{d}{dt}[\begin{array}{c} \nu_{E}(t)\\ \nu_{PV}(t)\\ \nu_{SOM}(t) \end{array}]=\frac{1}{0.02}[\begin{array}{ccc} 4 & -5 & -4\\ 5 & -5 & -2.05\\ 4 & -Q_{PV\to SOM} & -1 \end{array}][\begin{array}{c} \nu_{E}(t)\\ \nu_{PV}(t)\\ \nu_{SOM}(t) \end{array}] \end{array}$$ (12)

where $Q_{PV\to SOM}>0$ represents the PV to SOM coupling.

In order to investigate the effect of a non-zero $Q_{PV\;to\;SOM}$, eigenvalues of the matrix


$$\begin{array}{c} Y=[\begin{array}{ccc} 4 & -5 & -4\\ 5 & -5 & -2.05\\ 4 & -Q_{PV\to SOM} & -1 \end{array}] \end{array}$$ (13)

need to be analyzed. The determinant of Y is $\frac{59Q_{PV\to SOM}-220}{5}$, and needs to be equal to the multiplication of its eigenvalues. Therefore, if $Q_{PV\to SOM}>\frac{220}{59}$, *Y* has at least one positive eigenvalue and so the solutions of Eq. 12 are asymptotically unstable. Notice that in the reference model, in the limit of strong PV→SOM coupling, the system admits a stable non-zero fixed point. Even if the linear model gives valuable basic information on fundamental mechanisms in the vicinity of the fixed point, it is interesting to notice that the system is indeed highly nonlinear. In order to avoid divergence in the linear model, we thus restrict our analysis to $Q_{PV\to SOM}<\frac{220}{59}$. Through an analytical examination provided in Appendix C in S1 Text, it turns out that the solutions (for $0<Q_{PV\to SOM}\leq 220/59$) are damped oscillations and as one increases $Q_{PV\to SOM}$, the oscillations exhibit stronger damping. The real part of the complex eigenvalues of $\frac{Y}{0.02}$ which represents the damping factor, can be seen as a function of $Q_{PV\to SOM}$ in Fig 6C.

Secondly, we wonder what effect adding a SOM → SOM coupling can have on the base linear model 10. To this aim, we need to investigate the eigenvalues of:


$$\begin{array}{c} L=[\begin{array}{ccc} 4 & -5 & -4\\ 5 & -5 & -2.05\\ 4 & 0 & -1-Q_{SOM\to SOM} \end{array}] \end{array}$$ (14)

where $Q_{SOM\to SOM}>0$ represents the SOM to SOM coupling. By the examinations provided in Appendix D in S1 Text, it turns out that for any $0<Q_{SOM\to SOM}$, the solutions of the linear system with matrix *L* are damping oscillations and as one increases $Q_{SOM\to SOM}$, the oscillations exhibit stronger damping. The real part of the complex eigenvalues of $\frac{L}{0.02}$, which represents the damping factor, can be seen as a function of $Q_{SOM\to SOM}$ in Fig 6C.

## Discussion

In this work, we presented a mean-field model that predicts the emergent dynamics of the corresponding E-PV-SOM spiking neural networks. First, the mean field model reproduces a large variety of experimental observations. Moreover, it provides predictions on network’s stability, on the role of interneurons density for oscillatory dynamics and on the role of network’s connectivity. Finally, the mean field model allowed analytical treatments to identify the circuit mechanism generating oscillations. Indeed, by employing linear stability analyses, we identified a robust mechanism of neural oscillation in E-PV-SOM networks, which emerges solely from the specific connectivity between SOM and PV interneurons. Remarkably, the structure of this connectivity matches anatomical observations of cell-type-specific synaptic organization.

We first designed a low-dimensional mean-field formulation of E-PV-SOM networks that accurately captures the population dynamics in the Canonical Microcircuit Network Oscillation (CAMINOS) model [42], recently introduced in the context of spiking networks with parameters calibrated from anatomical and electrophysiological data. We used the master equation formalism developed in [27] and extended it to E-PV-SOM spiking neural networks with synaptic delay (implementing the approach developed in [49]). Although this mean field model does not introduce a new methodology *per se*, the proposed E–PV–SOM mean-field formulation represents a substantial improvement over previous E/I models, as it can reproduce a wide range of experimental observations on the distinct roles of interneuron subtypes in shaping gamma oscillations and network stability. First, the mean-field model accurately reproduces the emergence of gamma oscillations around 40 Hz, with SOM cells firing 6–7 ms later than excitatory and PV neurons, consistent with experimental observations [53]. Second, the model captures key findings from optogenetic experiments in the primary visual cortex involving the suppression of SOM and PV neurons [33,54]. Importantly, our mean-field model, consistent with direct numerical simulations of the spiking network, demonstrates that PV interneurons primarily determine the oscillation frequency, while SOM interneurons play an essential role in shaping and sustaining the oscillatory amplitude. Furthermore, PV cells play a central role in maintaining network stability against hypersynchronous, epileptic-like dynamics. When PV activity is suppressed beyond a certain threshold (approximately 70%), the oscillatory state in the mean-field model loses stability, leading to a bifurcation toward a high-activity regime of about 500 Hz, which represents the mean-field counterpart of a hypersynchronous epileptic state.

Our mean-field model also predicts that the frequency of oscillations in the E-PV-SOM model can cover alpha, beta and gamma ranges when varying properly the density ratio between PV and SOM cells in the network. Accordingly, the model also predicts that interregional differences in the ratio between PV and SOM cells could account for the dominant frequencies observed in cortical power spectra: alpha/beta oscillations prevailing in higher-order regions such as the prefrontal cortex, and faster gamma oscillations dominating in lower-order sensory areas such as the visual cortex [3,4,52,55–59]. Thanks to its capacity to reproduce the emergent dynamics of biologically realistic E–PV–SOM spiking networks, this mean-field model can serve as a building block for larger-scale frameworks that explicitly incorporate distinct inhibitory cell types.

Such a framework opens also new possibilities for modeling the emergence of multiple rhythms in interconnected E–PV–SOM modules, where different interneuron classes may dominate distinct frequency bands while remaining dynamically coupled. In addition, this mean-field formulation could be employed to investigate the organization of oscillatory power within a single cortical column across layers, particularly given the well-documented gradients in PV and SOM cell densities [50]. Finally, since these gradients differ across species, our model could help predict interspecies variations in the population dynamics of cortical columns arising from distinct PV and SOM connectivity patterns and laminar distributions [60]. Our model shows that cell specific connectivity, in particular between SOM and PV cells, determines the emergence of oscillations, whose frequency depends on interneurons density. This result proposes that, in models of a cortical column with cell-specific connections [61], distinct oscillatory rhythms can coexist based on the fundamental connectivity based mechanism here identified. This would enable the regulation of multiple rhythms with specific cross-frequency coupling. Moreover, it is known that SOM cells, at variance with PV, typically target the distal dendritic tree. In our model we used homogeneous synaptic delays between cells, but our approach could be extended to study effective longer delays of SOM cells, thus mimicking their slower effect on postsynaptic neurons. In general, future works may employ this mean field model to study cell-specific synaptic time scales and their effect on oscillations.

To uncover the fundamental mechanism giving rise to oscillations in the E–PV–SOM model, we further simplified our mean-field formulation. We first removed the synaptic delay ($\tau_{D}=0$), since it is well known that $\tau_{D}>0$ represents a classical mechanism for generating gamma oscillations at a frequency proportional to $1/\tau_{D}$ [62]. We also removed the spike-frequency adaptation time scale, thereby reducing our mean-field model to a three-dimensional system of ordinary differential equations (ODEs) with nonlinear transfer functions that depend on the biophysical parameters of the network and the distinct properties of each cell type. Interestingly, this reduced mean field model still exhibits stable oscillations whose qualitative features closely match those of the original biologically realistic model. In particular, SOM cells always fire after E and PV neurons. Second, increasing either the PV → SOM or SOM → SOM coupling strength abolishes the oscillatory regime. These results demonstrate that the oscillations observed in our model do not rely on ad hoc temporal parameters such as synaptic timescales or adaptation, but rather constitute an intrinsic property of the three-population E–PV–SOM circuit. In our view, this results identifies a distinct oscillatory state based on a three population mechanism, distinct from the classical two population PING in E/I systems. The fact that oscillations appear also in absence of spike frequency adaptation, synaptic dynamics or delays is an important feature of this type of three population mechanism. In our framework, oscillations are strongly driven by the three population microcircuit connectivity. Indeed, the emergence of oscillations depends critically on the specific structural organization of the network, characterized by weak PV → SOM and SOM→→SOM coupling, as observed experimentally [43]. While this oscillatory state shares some essential feature of the two-population PING, it clearly differs from the ING model that produces higher frequency oscillations and relies on PV-PV interactions.

We further extended our reductionist approach by considering a linear mean-field model. This approach isolates the essential connectivity motif among E, PV, and SOM populations. In this linear formulation, the only parameters are the coupling weights, that is, the connection strengths between distinct neuronal populations. An important advantage of this model is its analytical tractability, which allows for a formal investigation of the circuit’s dynamical properties. We showed that this linear system can sustain oscillations in the absence of SOM → SOM and PV → SOM connections, corresponding to the canonical architecture observed in anatomical studies. Moreover, the system becomes progressively less prone to oscillations as the strength of SOM → SOM and PV → SOM coupling increases. Interestingly, we analytically demonstrate that in this framework SOM cells always exhibit delayed activity with respect to E and PV neurons. This is a remarkable result, as it demonstrates that the oscillations observed in our biologically realistic model, and most likely those emerging in cortical circuits of E-PV-SOM cells, arise from a specific mechanism driven by the asymmetric connectivity between distinct interneuron types, namely SOM and PV cells.

Another interneuron subtype that has recently received attention is the vasoactive intestinal peptide-expressing (VIP) [43,63]. In this work, we focused on the development of E–PV–SOM circuits to elucidate the oscillatory mechanisms that this fundamental microcircuit can generate. Nevertheless, our mean-field approach, and the subsequent reduction to a linear analytical model, can readily be extended to include VIP interneurons. Although such an extension goes beyond the scope of the present study, investigating the influence of VIP neurons on gamma oscillations represents a promising future direction, well-suited to the methodological framework established here.

An important limitation of our study is the point-neuron approximation. Distinct interneuron types are known to have different dendritic targeting and integration properties; for example, SOM cells primarily project to the distal dendrites of excitatory pyramidal cells, whereas PV cells target their perisomatic regions [50]. These morphological and integrative differences could be incorporated into our framework by introducing cell-type-specific synaptic delays or integration time constants. These differences may be important given the potential role of bursting excitatory neurons in generating gamma oscillations [19,21]. However, to highlight the fundamental structural role of connectivity in generating oscillations, we deliberately chose to exclude such additional temporal parameters in the present work. Future studies could extend our model in this direction to assess how dendritic integration and spatial structure further shape and modulate oscillatory dynamics.

We conclude by noting that our model can be employed to investigate the functional roles of distinct interneurons, for example in the context of predictive coding, as recently explored in [64]. First, it would be interesting to study the impact of our desynchronisation manipulation, as opposed to classical silencing approaches. This manipulation preserves the overall level of inhibition while disrupting cell-specific synchronisation, which may have important consequences for predictive coding. Moreover, it would be interesting to assess whether our mean-field models (complete and linear formulation) reproduces the results reported in [64]. This would allow to directly investigate cell-specific connectivity rules supporting predictive coding.

## Methods

### Spiking network

To model the dynamics of the neuronal membrane potential *V*(*t*), we employ the adaptive exponential integrate-and-fire (AdEx) model [44]. The governing equations are given by


$$\begin{array}{c} C\frac{dV(t)}{dt}=g_{L}(E_{L}-V(t))+g_{L}\Delta e^{\frac{V(t)-V_{T}}{\Delta }}-w(t)+I(t), \end{array}$$ (15)

$$\begin{array}{c} \tau_{w}\frac{dw(t)}{dt}=a\left(V(t)-E_{L}\right)-w(t)+b\sum_{j}\delta \left(t-t_{j}\right), \end{array}$$ (16)

where *C* denotes the membrane capacitance, *g*_*L*_ the leak conductance, *E*_*L*_ the resting potential, *V*_*T*_ the effective threshold, and Δ the slope factor controlling the sharpness of spike initiation. The current *I*(*t*) corresponds to the sum of all synaptic inputs. The variable *w*(*t*) represents the adaptation current, which contributes a negative feedback to the membrane vol*t*age. Each time a spike occurs at *t*_*j*_, *w* is increased by *b* due to the in*t*egration of the Dirac delta function $\delta (t-t_{j})$. The parameter *a* encodes the subthreshold adaptation factor, $\tau_{w}$ defines the adaptation time constant, and the spike threshold was set to $V_{T}+5\Delta$. After spiking, the membrane potential is reset to *V*_reset_ and held constant for a refractory period *T*_ref_.

Parameter values were chosen to reflect electrophysiological properties of excitatory pyramidal neurons, parvalbumin-expressing (PV) interneurons, and somatostatin-expressing (SOM) interneurons [50,65–67]. Notably, PV cells lack spike-frequency adaptation, whereas both excitatory and SOM cells exhibit it. Moreover, spike-frequency adaptation tends to be more pronounced in SOM cells compared to pyramidal regular-spiking cells [68–70]. The leak potential *E*_*L*_ was tuned across cell types to capture average firing rates reported in V1, with higher activity in PV cells and lower rates in SOM and pyramidal cells [71]. All other parameters were set to biologically realistic values within standard ranges. A complete list of parameters is provided in Table 1.

**Table 1:** Neurons’ parameters.

Parameter	E, PV, SOM
*V*_*T*_	-50 mV
Δ	2, 0.5, 2 mV
*T*_ref_	5 ms
$\tau_{w}$	500 ms
*a*	4, 0, 4 nS
*b*	50, 0, 80 pA
*C*	200 pF
*g*_*L*_	10 nS
*E*_*L*_	-65, -65, -55 mV
*V*_reset_	-65 mV

Synaptic interactions were modeled as conductance-based inputs of the form


$$\begin{array}{c} I_{\text{syn}}=g(t)\left(E_{\text{rev}}^{\left\{E,I\right\}}-V(t)\right), \end{array}$$ (17)

where *V*(*t*) is the postsynaptic potential and $E_{\text{rev}}^{\left\{E,I\right\}}$ is the reversal potential of excitatory (E) or inhibitory (I) synapses. The synaptic conductance *g*(*t*) evolves according to


$$\begin{array}{c} \tau^{\left\{E,I\right\}}\frac{dg}{dt}=-g(t)+Q\sum_{j}\delta (t-t_{j}-\tau_{d}), \end{array}$$ (18)

where $\tau^{\left\{E,I\right\}}$ is the excitatory (E) or inhibitory (I) synaptic decay time constant, $\tau_{d}$ the synaptic delay, $\delta (t-t_{j}-\tau_{d})$ representing a presynaptic spike arriving at the delayed time, and *Q* the quantal conductance (denoted $Q_{X\to Y}$ for connections from population *X* to *Y*). Synaptic strengths were set based on experimental estimates [72]. A complete list of synaptic parameters is provided in Table 2.

**Table 2:** Synaptic parameters.

Parameter	Value
$\tau_{d}$	2 ms
$\tau^{E}$	2 ms
$\tau^{I}$	8 ms
$E_{\text{rev}}^{E}$	0 mV
$E_{\text{rev}}^{I}$	-80 mV
$Q_{\text{\{PV,SOM\}}}\to \text{\{E,PV\}}$	5 nS
$Q_{\text{E}\to \text{E}}$	2 nS
$Q_{\text{E}\to \text{\{PV,SOM\}}}$	3 nS
$Q_{\text{Ext}\to \text{\{E,PV\}}}$	2.5 nS

The network consisted of $N_{E}=8000$ excitatory pyramidal neurons and $N_{I}=1500$ inhibitory interneurons, equally divided into PV and SOM subpopulations ($N_{PV}=750$, $N_{SOM}=750$). Connectivity was random and sparse: any presynaptic neuron formed a synapse onto a postsynaptic target with probability *p* = 0.07, an average value across cell types estimated according to experimental observations [73]. Notice that cell-type specific level of connectivity is here neglected, apart from the absence of recurrent SOM-to-SOM and PV-to-SOM connections. Future works may extend this model to include cell-specific levels of connectivity, which will surely affect that stability of oscillations (see for example the bifurcation diagram in function of *p* in Fig 1E). In the reference model (Fig 1A), two connections are absent in line with experimental evidence: PV → SOM and SOM → SOM [43]. Thus, apart from the analyses of Section 5, we set $Q_{PV\to SOM}=0$ and $Q_{SOM\to SOM}=0$.

External drive was modeled as independent Poisson spike trains at 4 Hz, generated by $p\cdot N_{E}$ excitatory neurons, each providing inputs with quantal conductance *Q*_Ext_ = 2.5 nS to excitatory and PV cells. The choice of 4Hz allows to set realistic baseline average firing rates of E, SOM and PV cells as in experimental data, see [42]. Let us notice that increasing the input increases also oscillations’ frequency but does not change the qualitative features of the system, such as the role of PV/SOM ratio, SOM/PV silencing and network stability in function of PV to SOM and SOM to SOM connections. Population activity for each cell class was calculated as


$$\begin{array}{c} \nu (t)=\frac{1}{\Delta t}\frac{n_{act}(t,t+\Delta t)}{N}, \end{array}$$ (19)

where *N* is the size of the population and $n_{act}(t,t+\Delta t)$ the number of spikes in the interval (t,t+Δt). Unless otherwise stated, we used Δt=0.1 ms. Resulting activity traces ν(t) were convolved with a Gaussian kernel of width 2 ms for visualization.

Simulations were implemented in Python using the Brian2 package [74]. Differential equations were integrated using the Euler method with a timestep of *dt* = 0.1 *ms*.

### Transfer function derivation for the mean-field

For the mean-field description, we derived transfer functions ${ℱ}_{E},{ℱ}_{PV},{ℱ}_{SOM}$ that map presynaptic firing rates onto the output rate of each neuron type. The approach follows the shot-noise diffusion approximation [27,47,75], in which the effective input statistics are estimated and then converted into an output rate through a fluctuation-dependent threshold.

#### Input statistics.

Given presynaptic rates $\nu_{E},\nu_{PV},\nu_{SOM},\nu_{\text{ext}}$, the corresponding event rates are


$$\begin{array}{c} f_{E}=\nu_{E}pN_{E},\;f_{PV}=\nu_{PV}pN_{PV},\;f_{SOM}=\nu_{SOM}pN_{SOM},\;f_{\text{ext}}=\nu_{\text{ext}}pN_{E}, \end{array}$$

with connection probability *p* and population sizes *N*_*X*_. Each input contributes a mean synaptic conductance $\mu G_{X}=Q_{X}\tau_{X}f_{X}$. The total conductance is


$$\begin{array}{c} \mu_{G}=g_{L}+\mu G_{E}+\mu G_{PV}+\mu G_{SOM}+\mu G_{\text{ext}}, \end{array}$$

yielding an effective time constant $T_{m}=C_{m}/\mu_{G}$. The mean subthreshold potential is approximated as


$$\begin{array}{c} \mu_{V}=\frac{\mu G_{E}E_{E}+\mu G_{\text{ext}}E_{E}+(\mu G_{PV}+\mu G_{SOM})E_{I}+g_{L}E_{L}-w}{\mu_{G}}, \end{array}$$

with *w* = 0 for PV cells.

#### Voltage fluctuations.

The postsynaptic impact of a single event of type *X* is


$$\begin{array}{c} U_{X}=\frac{Q_{X}}{\mu_{G}}(E_{X}-\mu_{V}), \end{array}$$

where *E*_*X*_ is the corresponding reversal potential. The variance of membrane potential fluctuations is


$$\begin{array}{c} \sigma_{V}^{2}=\sum_{X}f_{X}\;\frac{(U_{X}\tau_{X}{)}^{2}}{2(\tau_{X}+T_{m})}, \end{array}$$

and the effective correlation time is


$$\begin{array}{c} T_{v}=\frac{\sum_{X}f_{X}(U_{X}\tau_{X}{)}^{2}}{\sum_{X}\frac{f_{X}(U_{X}\tau_{X}{)}^{2}}{\tau_{X}+T_{m}}},\;T_{v}^{N}=\frac{g_{L}}{C_{m}}T_{v}. \end{array}$$

#### Effective threshold and transfer function.

To capture nonlinear dependence on input statistics, the effective threshold is expressed as a second-order polynomial in the normalized variables $(\mu_{V}-\mu_{V0})/D\mu_{V0}$, $(\sigma_{V}-\sigma_{V0})/D\sigma_{0}$, and $(T_{v}^{N}-T_{v0}^{N})/DT_{v0}^{N}$, with coefficients $P_{0},\ldots ,P_{9}$ fitted from AdEx single-cell simulations. Fitted values were estimated by minimizing a least-squares cost function defined as the squared relative error $((\nu_{pred}-\nu_{obs})/\nu_{obs}{)}^{2}$ using the Nelder–Mead simplex algorithm. We observed that increasing the polynomial order did not improve the fitting performance. All numerical parameters and fitted coefficients are reported in Tables 3 and 4. The output firing rate is then


$$\begin{array}{c} \nu_{\text{out}}=\frac{g_{L}}{2C_{m}T_{v}^{N}}\;erfc\;\left(\frac{V_{thr}^{eff}-\mu_{V}}{\sqrt{2}\;\sigma_{V}}\right). \end{array}$$

**Table 3:** Fitted parameters for each cell type.

Cell type	*P*_0_	$P_{\mu_{V}}$	$P_{\sigma_{V}}$	$P_{\tau_{V}^{N}}$	$P_{\mu_{V}^{2}}$	$P_{\sigma_{V}^{2}}$	$P_{(\tau_{V}^{N}{)}^{2}}$	$P_{\mu_{V}\sigma_{V}}$	$P_{\mu_{V}\tau_{V}^{N}}$	$P_{\sigma_{V}\tau_{V}^{N}}$
E	-0.049734	0.007999	0.009232	-0.002773	0.027645	-0.037666	0.029874	-0.023012	-0.005805	0.022400
PV	-0.052335	0.005472	-0.010339	-0.014467	-0.000929	0.000323	0.002562	0.023115	-0.023466	-0.034865
SOM	-0.046763	0.000063	-0.003225	-0.011496	0.000968	0.001398	0.009397	0.013090	0.022406	0.010788

**Table 4:** Baseline fit parameters for each cell type.

Cell Type	$\mu_{V0}$	$D\mu_{V0}$	$s_{V0}$	$Ds_{V0}$	$T_{vN0}$	$DT_{vN0}$
E	-0.06	0.05	0.004	0.006	0.5	1
PV	-0.06	0.01	0.004	0.006	0.5	1
SOM	-0.05	0.01	0.004	0.006	0.5	1

### Fixed point computation and stability

The fixed points of the reference mean-field model (Eq. 1) were obtained by imposing the steady-state condition in which all time derivatives vanish. Under this condition, the system reduces to a set of nonlinear algebraic equations:


$$\begin{array}{cc} \nu^{E} & ={ℱ}_{E}(\nu^{E},\nu^{PV},\nu^{SOM},w^{E}),\\ \nu^{PV} & ={ℱ}_{PV}(\nu^{E},\nu^{PV},\nu^{SOM}),\\ \nu^{SOM} & ={ℱ}_{SOM}(\nu^{E},\nu^{PV},\nu^{SOM},w^{SOM}),\\ w^{E} & =\tau_{w}\left[b_{E}\nu^{E}+a_{E}(\mu_{v}^{E}(\nu^{E},\nu^{PV},\nu^{SOM},w^{E})-E_{l}^{E})\right],\\ w^{SOM} & =\tau_{w}\left[b_{SOM}\nu^{SOM}+a_{SOM}(\mu_{v}^{SOM}(\nu^{E},\nu^{PV},\nu^{SOM},w^{SOM})-E_{l}^{SOM})\right]. \end{array}$$ (20)

We reformulated the problem as a root-finding task. The nonlinear system was solved numerically using a multidimensional root-finding algorithm (SciPy root), initialized from a biologically plausible initial condition. Convergence was assessed using the default tolerance criteria of the solver. The resulting solution corresponds to a fixed point. We have assessed the stability of the fixed points numerically. In practice, starting from the fixed-point solution, we introduced a small perturbation and observed the resulting trajectory. We then estimated the maximum and minimum values of $\nu^{E}(t)$ to construct a phase diagram (see Fig 1E for an example). Future works may perform more rigorous analysis in this delayed ODE system, by imposing for example the condition of marginal stability, assuming an eigenvalue of the form λ=iω.

## Supporting information

S1 TextSupporting information.Appendix A: Details of Proof 1 (linear model). Appendix B: Eigenvalues of the matrix *F* (linear model). Appendix C: Solutions in the linear model as a function of $Q_{PV\to SOM}$. Appendix D: Solutions in the linear model as a function of $Q_{SOM\to SOM}$.(PDF)

## References

[1] Buzsáki G. Rhythms of the brain. USA: Oxford University Press; 2006.

[2] Wang H-P, Spencer D, Fellous J-M, Sejnowski TJ. Synchrony of thalamocortical inputs maximizes cortical reliability. Science. 2010;328(5974):106–9. doi: 10.1126/science.1183108 20360111 PMC2859205

[3] Vinck M, Uran C, Spyropoulos G, Onorato I, Broggini AC, Schneider M, et al. Principles of large-scale neural interactions. Neuron. 2023;111(7):987–1002. doi: 10.1016/j.neuron.2023.03.015 37023720

[4] Vinck M, Uran C, Dowdall JR, Rummell B, Canales-Johnson A. Large-scale interactions in predictive processing: oscillatory versus transient dynamics. Trends Cogn Sci. 2025;29(2):133–48. doi: 10.1016/j.tics.2024.09.013 39424521 PMC7616854

[5] Fries P. A mechanism for cognitive dynamics: neuronal communication through neuronal coherence. Trends Cogn Sci. 2005;9(10):474–80. doi: 10.1016/j.tics.2005.08.011 16150631

[6] Bressler SL, Kelso JAS. Cortical coordination dynamics and cognition. Trends Cogn Sci. 2001;5(1):26–36. doi: 10.1016/s1364-6613(00)01564-3 11164733

[7] Varela F, Lachaux JP, Rodriguez E, Martinerie J. The brainweb: phase synchronization and large-scale integration. Nat Rev Neurosci. 2001;2(4):229–39. doi: 10.1038/35067550 11283746

[8] Buzsáki G, Anastassiou CA, Koch C. The origin of extracellular fields and currents--EEG, ECoG, LFP and spikes. Nat Rev Neurosci. 2012;13(6):407–20. doi: 10.1038/nrn3241 22595786 PMC4907333

[9] Brunel N. Dynamics of sparsely connected networks of excitatory and inhibitory spiking neurons. J Comput Neurosci. 2000;8(3):183–208. doi: 10.1023/a:1008925309027 10809012

[10] Tiesinga P, Sejnowski TJ. Cortical enlightenment: are attentional gamma oscillations driven by ING or PING? Neuron. 2009;63:727–32. 19778503 10.1016/j.neuron.2009.09.009 PMC2778762

[11] Börgers C, Kopell N. Synchronization in networks of excitatory and inhibitory neurons with sparse, random connectivity. Neural Comput. 2003;15(3):509–38. doi: 10.1162/089976603321192059 12620157

[12] Traub R. Fast oscillations. Scholarpedia. 2006;1(12):1764. doi: 10.4249/scholarpedia.1764

[13] Jadi MP, Sejnowski TJ. Cortical oscillations arise from contextual interactions that regulate sparse coding. Proc Natl Acad Sci U S A. 2014;111(18):6780–5. doi: 10.1073/pnas.1405300111 24742427 PMC4020078

[14] Wilson HR, Cowan JD. Excitatory and inhibitory interactions in localized populations of model neurons. Biophys J. 1972;12(1):1–24. doi: 10.1016/S0006-3495(72)86068-5 4332108 PMC1484078

[15] Hasenstaub A, Shu Y, Haider B, Kraushaar U, Duque A, McCormick DA. Inhibitory postsynaptic potentials carry synchronized frequency information in active cortical networks. Neuron. 2005;47(3):423–35. doi: 10.1016/j.neuron.2005.06.016 16055065

[16] Csicsvari J, Jamieson B, Wise KD, Buzsáki G. Mechanisms of gamma oscillations in the hippocampus of the behaving rat. Neuron. 2003;37(2):311–22. doi: 10.1016/s0896-6273(02)01169-8 12546825

[17] Buzsáki G, Wang X-J. Mechanisms of gamma oscillations. Annu Rev Neurosci. 2012;35:203–25. doi: 10.1146/annurev-neuro-062111-150444 22443509 PMC4049541

[18] Le Van Quyen M, Muller LE 2nd, Telenczuk B, Halgren E, Cash S, Hatsopoulos NG, et al. High-frequency oscillations in human and monkey neocortex during the wake-sleep cycle. Proc Natl Acad Sci U S A. 2016;113(33):9363–8. doi: 10.1073/pnas.1523583113 27482084 PMC4995938

[19] Onorato I, Tzanou A, Schneider M, Uran C, Broggini AC, Vinck M. Distinct roles of PV and Sst interneurons in visually induced gamma oscillations. Cell Rep. 2025;44(3):115385. doi: 10.1016/j.celrep.2025.115385 40048428

[20] Vinck M, Womelsdorf T, Fries P. Gamma-band synchronization and information transmission. In: Quiroga-Quian R, Panzeri S, editors. Principles of neural coding. CRC Press; 2013.

[21] Onorato I, Neuenschwander S, Hoy J, Lima B, Rocha K-S, Broggini AC, et al. A distinct class of bursting neurons with strong gamma synchronization and stimulus selectivity in monkey V1. Neuron. 2020;105(1):180-197.e5. doi: 10.1016/j.neuron.2019.09.039 31732258

[22] Cardin JA, Carlén M, Meletis K, Knoblich U, Zhang F, Deisseroth K, et al. Driving fast-spiking cells induces gamma rhythm and controls sensory responses. Nature. 2009;459(7247):663–7. doi: 10.1038/nature08002 19396156 PMC3655711

[23] Vinck M, Bos JJ, Van Mourik-Donga LA, Oplaat KT, Klein GA, Jackson JC, et al. Cell-type and state-dependent synchronization among rodent somatosensory, visual, perirhinal cortex, and hippocampus CA1. Front Syst Neurosci. 2016;9:187. doi: 10.3389/fnsys.2015.00187 26834582 PMC4722130

[24] Montbrió E, Pazó D, Roxin A. Macroscopic description for networks of spiking neurons. Phys Rev X. 2015;5(2):021028. doi: 10.1103/physrevx.5.021028

[25] di Volo M, Torcini A. Transition from asynchronous to oscillatory dynamics in balanced spiking networks with instantaneous synapses. Phys Rev Lett. 2018;121(12):128301. doi: 10.1103/PhysRevLett.121.128301 30296134

[26] Goldobin DS, di Volo M, Torcini A. Reduction methodology for fluctuation driven population dynamics. Phys Rev Lett. 2021;127(3):038301. doi: 10.1103/PhysRevLett.127.038301 34328756

[27] Volo M di, Romagnoni A, Capone C, Destexhe A. Biologically realistic mean-field models of conductance-based networks of spiking neurons with adaptation. Neural Comput. 2019;31(4):653–80. doi: 10.1162/neco_a_01173 30764741

[28] Batista-Brito R, Zagha E, Ratliff JM, Vinck M. Modulation of cortical circuits by top-down processing and arousal state in health and disease. Curr Opin Neurobiol. 2018;52:172–81. doi: 10.1016/j.conb.2018.06.008 30064117

[29] Freund TF. Interneuron diversity series: rhythm and mood in perisomatic inhibition. Trends Neurosci. 2003;26(9):489–95. doi: 10.1016/S0166-2236(03)00227-3 12948660

[30] Moore CI, Carlen M, Knoblich U, Cardin JA. Neocortical interneurons: from diversity, strength. Cell. 2010;142:184–8. 20655460 10.1016/j.cell.2010.07.005 PMC3655709

[31] Cardin JA. Inhibitory interneurons regulate temporal precision and correlations in cortical circuits. Trends Neurosci. 2018;41(10):689–700. doi: 10.1016/j.tins.2018.07.015 30274604 PMC6173199

[32] Miri ML, Vinck M, Pant R, Cardin JA. Altered hippocampal interneuron activity precedes ictal onset. Elife. 2018;7:e40750. doi: 10.7554/eLife.40750 30387711 PMC6245730

[33] Veit J, Hakim R, Jadi MP, Sejnowski TJ, Adesnik H. Cortical gamma band synchronization through somatostatin interneurons. Nat Neurosci. 2017;20(7):951–9. doi: 10.1038/nn.4562 28481348 PMC5511041

[34] Chen G, Zhang Y, Li X, Zhao X, Ye Q, Lin Y, et al. Distinct inhibitory circuits orchestrate cortical beta and gamma band oscillations. Neuron. 2017;96(6):1403-1418.e6. doi: 10.1016/j.neuron.2017.11.033 29268099 PMC5864125

[35] Lee JH, Whittington MA, Kopell NJ. Top-down beta rhythms support selective attention via interlaminar interaction: a model. PLoS Comput Biol. 2013;9(8):e1003164. doi: 10.1371/journal.pcbi.1003164 23950699 PMC3738471

[36] Moreni G, Zou L, Pennartz CMA, Mejias JF. Synaptic plasticity facilitates oscillations in a V1 cortical column model with multiple interneuron types. Front Comput Neurosci. 2025;19:1568143. doi: 10.3389/fncom.2025.1568143 40370493 PMC12075120

[37] Parker MM, Rubin JE, Huang C. State modulation in spatial networks with three interneuron subtypes. Sci Adv. 2025;11(26):eads9134. doi: 10.1126/sciadv.ads9134 40561011 PMC13108820

[38] Milea D, Meneghetti N, Mazzoni A, Cataldo E. A spiking lif model captures the role of somatostatin and parvalbumin neurons in generating oscillations in v1. bioRxiv. 2025:2025–01.

[39] Wendling F, Koksal-Ersoz E, Al-Harrach M, Yochum M, Merlet I, Ruffini G, et al. Multiscale neuro-inspired models for interpretation of EEG signals in patients with epilepsy. Clin Neurophysiol. 2024;161:198–210. doi: 10.1016/j.clinph.2024.03.006 38520800

[40] Hahn G, Kumar A, Schmidt H, Knösche TR, Deco G. Rate and oscillatory switching dynamics of a multilayer visual microcircuit model. Elife. 2022;11:e77594. doi: 10.7554/eLife.77594 35994330 PMC9395191

[41] Sanchez-Todo R, Bastos AM, Lopez-Sola E, Mercadal B, Santarnecchi E, Miller EK, et al. A physical neural mass model framework for the analysis of oscillatory generators from laminar electrophysiological recordings. Neuroimage. 2023;270:119938. doi: 10.1016/j.neuroimage.2023.119938 36775081 PMC12631363

[42] Tahvili F, Vinck M, di Volo M. PV and SOM cells play distinct causal roles in controlling network oscillations and stability. Cell Rep. 2025;44(8):116131. doi: 10.1016/j.celrep.2025.116131 40783942

[43] Pfeffer CK, Xue M, He M, Huang ZJ, Scanziani M. Inhibition of inhibition in visual cortex: the logic of connections between molecularly distinct interneurons. Nat Neurosci. 2013;16(8):1068–76. doi: 10.1038/nn.3446 23817549 PMC3729586

[44] Brette R, Gerstner W. Adaptive exponential integrate-and-fire model as an effective description of neuronal activity. J Neurophysiol. 2005;94(5):3637–42. doi: 10.1152/jn.00686.2005 16014787

[45] Spyropoulos G, Saponati M, Dowdall JR, Schölvinck ML, Bosman CA, Lima B, et al. Spontaneous variability in gamma dynamics described by a damped harmonic oscillator driven by noise. Nat Commun. 2022;13(1):2019. doi: 10.1038/s41467-022-29674-x 35440540 PMC9018758

[46] El Boustani S, Marre O, Béhuret S, Baudot P, Yger P, Bal T, et al. Network-state modulation of power-law frequency-scaling in visual cortical neurons. PLoS Comput Biol. 2009;5(9):e1000519. doi: 10.1371/journal.pcbi.1000519 19779556 PMC2740863

[47] Zerlaut Y, Teleńczuk B, Deleuze C, Bal T, Ouanounou G, Destexhe A. Heterogeneous firing rate response of mouse layer V pyramidal neurons in the fluctuation-driven regime. J Physiol. 2016;594(13):3791–808. doi: 10.1113/JP272317 27146816 PMC4929333

[48] Destexhe A, Rudolph M, Paré D. The high-conductance state of neocortical neurons in vivo. Nat Rev Neurosci. 2003;4(9):739–51. doi: 10.1038/nrn1198 12951566

[49] Tahvili F, Destexhe A. A mean-field model of gamma-frequency oscillations in networks of excitatory and inhibitory neurons. J Comput Neurosci. 2024;52(2):165–81. doi: 10.1007/s10827-024-00867-1 38512693

[50] Tremblay R, Lee S, Rudy B. GABAergic interneurons in the neocortex: from cellular properties to circuits. Neuron. 2016;91(2):260–92. doi: 10.1016/j.neuron.2016.06.033 27477017 PMC4980915

[51] Kim Y, Yang GR, Pradhan K, Venkataraju KU, Bota M, García Del Molino LC, et al. Brain-wide maps reveal stereotyped cell-type-based cortical architecture and subcortical sexual dimorphism. Cell. 2017;171(2):456-469.e22. doi: 10.1016/j.cell.2017.09.020 28985566 PMC5870827

[52] Murray JD, Bernacchia A, Freedman DJ, Romo R, Wallis JD, Cai X, et al. A hierarchy of intrinsic timescales across primate cortex. Nat Neurosci. 2014;17(12):1661–3. doi: 10.1038/nn.3862 25383900 PMC4241138

[53] Onorato I, Neuenschwander S, Hoy J, Lima B, Rocha K-S, Broggini AC, et al. A distinct class of bursting neurons with strong gamma synchronization and stimulus selectivity in monkey V1. Neuron. 2020;105(1):180-197.e5. doi: 10.1016/j.neuron.2019.09.039 31732258

[54] Chen G, Zhang Y, Li X, Zhao X, Ye Q, Lin Y, et al. Distinct inhibitory circuits orchestrate cortical beta and gamma band oscillations. Neuron. 2017;96:1403-1418.e6. 10.1016/j.neuron.2017.11.033 PMC5864125 29268099

[55] Bastos AM, Vezoli J, Bosman CA, Schoffelen J-M, Oostenveld R, Dowdall JR, et al. Visual areas exert feedforward and feedback influences through distinct frequency channels. Neuron. 2015;85(2):390–401. doi: 10.1016/j.neuron.2014.12.018 25556836

[56] Vezoli J, Vinck M, Bosman CA, Bastos AM, Lewis CM, Kennedy H, et al. The role of anatomical connection strength for interareal communication in macaque cortex; 2020. Available from: https://ssrn.com/abstract=3751057

[57] Vezoli J, Magrou L, Goebel R, Wang X-J, Knoblauch K, Vinck M, et al. Cortical hierarchy, dual counterstream architecture and the importance of top-down generative networks. Neuroimage. 2021;225:117479. doi: 10.1016/j.neuroimage.2020.117479 33099005 PMC8244994

[58] Honey CJ, Thesen T, Donner TH, Silbert LJ, Carlson CE, Devinsky O, et al. Slow cortical dynamics and the accumulation of information over long timescales. Neuron. 2012;76(2):423–34. doi: 10.1016/j.neuron.2012.08.011 23083743 PMC3517908

[59] Buffalo EA, Fries P, Landman R, Buschman TJ, Desimone R. Laminar differences in gamma and alpha coherence in the ventral stream. Proc Natl Acad Sci U S A. 2011;108(27):11262–7. doi: 10.1073/pnas.1011284108 21690410 PMC3131344

[60] Medalla M, Mo B, Nasar R, Zhou Y, Park J, Luebke JI. Comparative features of calretinin, calbindin, and parvalbumin expressing interneurons in mouse and monkey primary visual and frontal cortices. J Comp Neurol. 2023;531(18):1934–62. doi: 10.1002/cne.25514 37357562 PMC10749991

[61] Binzegger T, Douglas RJ, Martin KAC. A quantitative map of the circuit of cat primary visual cortex. J Neurosci. 2004;24(39):8441–53. doi: 10.1523/JNEUROSCI.1400-04.2004 15456817 PMC6729898

[62] Brunel N, Wang X-J. What determines the frequency of fast network oscillations with irregular neural discharges? I. Synaptic dynamics and excitation-inhibition balance. J Neurophysiol. 2003;90(1):415–30. doi: 10.1152/jn.01095.2002 12611969

[63] Veit J, Handy G, Mossing DP, Doiron B, Adesnik H. Cortical VIP neurons locally control the gain but globally control the coherence of gamma band rhythms. Neuron. 2023;111(3):405-417.e5. doi: 10.1016/j.neuron.2022.10.036 36384143 PMC9898108

[64] Lee K, Pennartz CMA, Mejias JF. Cortical networks with multiple interneuron types generate oscillatory patterns during predictive coding. PLoS Comput Biol. 2025;21(9):e1013469. doi: 10.1371/journal.pcbi.1013469 40929236 PMC12443261

[65] McCormick DA, Connors BW, Lighthall JW, Prince DA. Comparative electrophysiology of pyramidal and sparsely spiny stellate neurons of the neocortex. J Neurophysiol. 1985;54(4):782–806. doi: 10.1152/jn.1985.54.4.782 2999347

[66] Kawaguchi Y, Kubota Y. Physiological and morphological identification of somatostatin- or vasoactive intestinal polypeptide-containing cells among GABAergic cell subtypes in rat frontal cortex. J Neurosci. 1996;16(8):2701–15. doi: 10.1523/JNEUROSCI.16-08-02701.1996 8786446 PMC6578756

[67] McGarry LM, Packer AM, Fino E, Nikolenko V, Sippy T, Yuste R. Quantitative classification of somatostatin-positive neocortical interneurons identifies three interneuron subtypes. Front Neural Circuits. 2010;4:12. doi: 10.3389/fncir.2010.00012 20617186 PMC2896209

[68] Romero-Sosa JL, Motanis H, Buonomano DV. Differential excitability of PV and SST neurons results in distinct functional roles in inhibition stabilization of up states. J Neurosci. 2021;41(34):7182–96. doi: 10.1523/JNEUROSCI.2830-20.2021 34253625 PMC8387123

[69] Yavorska I, Wehr M. Somatostatin-expressing inhibitory interneurons in cortical circuits. Front Neural Circuits. 2016;10:76. doi: 10.3389/fncir.2016.00076 27746722 PMC5040712

[70] Erisir A, Lau D, Rudy B, Leonard CS. Function of specific K(+) channels in sustained high-frequency firing of fast-spiking neocortical interneurons. J Neurophysiol. 1999;82(5):2476–89. doi: 10.1152/jn.1999.82.5.2476 10561420

[71] Ma W, Liu B, Li Y, Huang ZJ, Zhang LI, Tao HW. Visual representations by cortical somatostatin inhibitory neurons--selective but with weak and delayed responses. J Neurosci. 2010;30(43):14371–9. doi: 10.1523/JNEUROSCI.3248-10.2010 20980594 PMC3001391

[72] Destexhe A, Mainen ZF, Sejnowski TJ. Kinetic models of synaptic transmission. Methods Neuronal Model. 1998;2:1–25.

[73] Campagnola L, Seeman SC, Chartrand T, Kim L, Hoggarth A, Gamlin C, et al. Local connectivity and synaptic dynamics in mouse and human neocortex. Science. 2022;375(6585):eabj5861. doi: 10.1126/science.abj5861 35271334 PMC9970277

[74] Stimberg M, Brette R, Goodman DF. Brian 2, an intuitive and efficient neural simulator. Elife. 2019;8:e47314. doi: 10.7554/eLife.47314 31429824 PMC6786860

[75] Fourcaud N, Brunel N. Dynamics of the firing probability of noisy integrate-and-fire neurons. Neural Comput. 2002;14(9):2057–110. doi: 10.1162/089976602320264015 12184844

