# Supplementary material for: A mean-field model of neural networks with PV and SOM interneurons reveals connectivity-based mechanisms of gamma oscillations
Source: PLoS Comput Biol. 2026 Jun 10;22(6):e1014378. doi: 10.1371/journal.pcbi.1014378 (PMC13286281; doi:10.1371/journal.pcbi.1014378)
Supplement: S1 Text — Appendix A: Details of Proof 1 (linear model). Appendix B: Eigenvalues of the matrix F (linear model). Appendix C: Solutions in the linear model as a function of QPV→SOM. Appendix D: Solutions in the linear model as a function of QSOM→SOM. (PDF) [file pcbi.1014378.s001.pdf]

Appendix A

Proof 1. *Suppose that P is a strictly positive diagonal $3*3$ matrix like $\text{diag}(a1, a2, a3)$ and $M = PAP^{-1}$. The eigenvalues of M are the same as those of A and if x is an eigenvector of A then Px is an eigenvector of M. The eigenvector of M corresponding to the eigenvalue $\alpha + i\omega$ is $\begin{bmatrix} a_1x_1 & a_2x_2 & a_3x_3 \end{bmatrix}^T$. Since a_i are strictly positive numbers, the phase of a_ix_i is the same as the phase of x_i and therefore it is enough to prove for M.*

We take $P = \text{diag}(1, Q_{PV\,to\,E}, Q_{SOM\,to\,E})$ and therefore

$$M = \begin{bmatrix} Q_{E\,to\,E} - 1 & -1 & -1 \\ Q_{E\,to\,PV}Q_{PV\,to\,E} & -Q_{PV\,to\,PV} - 1 & -\frac{Q_{SOM\,to\,PV}Q_{PV\,to\,E}}{Q_{SOM\,to\,E}} \\ Q_{E\,to\,SOM}Q_{SOM\,to\,E} & 0 & -1 \end{bmatrix}.$$

Without loss of generality, suppose that the eigenvector of $\alpha + i\omega$ is $\begin{bmatrix} \nu_1 & \nu_2 & 1 \end{bmatrix}^T$ and, therefore,

$$\begin{bmatrix} Q_{E\,to\,E} - 1 & -1 & -1 \\ Q_{E\,to\,PV}Q_{PV\,to\,E} & -Q_{PV\,to\,PV} - 1 & -\frac{Q_{SOM\,to\,PV}Q_{PV\,to\,E}}{Q_{SOM\,to\,E}} \\ Q_{E\,to\,SOM}Q_{SOM\,to\,E} & 0 & -1 \end{bmatrix} \begin{bmatrix} \nu_1 \\ \nu_2 \\ 1 \end{bmatrix} = (\alpha + i\omega) \begin{bmatrix} \nu_1 \\ \nu_2 \\ 1 \end{bmatrix}.$$

From the third row, $\nu_1 = \frac{1+\alpha}{Q_{E\,to\,SOM}Q_{SOM\,to\,E}} + \frac{\omega}{Q_{E\,to\,SOM}Q_{SOM\,to\,E}}i$ is derived. Since, ω , $Q_{E\,to\,SOM}$ and $Q_{SOM\,to\,E}$ are larger than zero, we have $\frac{\omega}{Q_{E\,to\,SOM}Q_{SOM\,to\,E}} > 0$ and therefore E activity has a phase advance to the SOM activity.

From the first row, we have $(Q_{E\,to\,E} - 1)\nu_1 - \nu_2 - 1 = (\alpha + i\omega)\nu_1$ and it follows that $\nu_2 = ((Q_{E\,to\,E} - 1) - \alpha - i\omega)\nu_1 - 1$. By substituting $\nu_1 = \frac{1+\alpha}{Q_{E\,to\,SOM}Q_{SOM\,to\,E}} + \frac{\omega}{Q_{E\,to\,SOM}Q_{SOM\,to\,E}}i$, we conclude that the imaginary part of ν_2 is

$$\Im(\nu_2) = \frac{((Q_{E\,to\,E} - 1) - 1 - 2\alpha)\omega}{Q_{E\,to\,SOM}Q_{SOM\,to\,E}}.$$

Now it remains to prove that this imaginary part is strictly positive. To do this, it is enough to prove that $Q_{E\,to\,E} - 1 > 1 + 2\alpha$. Suppose, for the sake of contradiction, that $K = (-(Q_{E\,to\,E} - 1) + 1 + 2\alpha) \geq 0$. We already knew that the matrix A, consequently M, has three eigenvalues $\alpha + i\omega$, $\alpha - i\omega$ and r. It is known that the sum of eigenvalues is equal to the sum of diagonal entries and therefore $r = (Q_{E\,to\,E} - 1) - (Q_{PV\,to\,PV} + 1) - (1 + 2\alpha)$. On the other hand, since r is an eigenvalue of M then $\det(M - rI) = 0$ and it follows that

$$\begin{vmatrix} Q_{E\,to\,E} - 1 - r & -1 & -1 \\ Q_{E\,to\,PV}Q_{PV\,to\,E} & -Q_{PV\,to\,PV} - 1 - r & -\frac{Q_{SOM\,to\,PV}Q_{PV\,to\,E}}{Q_{SOM\,to\,E}} \\ Q_{E\,to\,SOM}Q_{SOM\,to\,E} & 0 & -1 - r \end{vmatrix} = 0.$$

Expanding the determinant along its third row and taking $K = (-(Q_{E\,to\,E} - 1) + 1 + 2\alpha)$ results in $\det(M - rI) = Q_{PV\,to\,E}Q_{SOM\,to\,PV}Q_{E\,to\,SOM} + Q_{E\,to\,SOM}Q_{SOM\,to\,E}K + (K + Q_{PV\,to\,PV})(Q_{E\,to\,PV}Q_{PV\,to\,E} + K(Q_{PV\,to\,PV} + 2 + 2\alpha)$. On the other hand from $Q_{E\,to\,E} > 0$ and $K \geq 0$ we have $2 + 2\alpha > 0$. $\det(M - rI)$ is a sum of positive terms and some of these terms are strictly positive (like $Q_{PV\,to\,E}Q_{SOM\,to\,PV}Q_{E\,to\,SOM}$, $Q_{PV\,to\,PV}Q_{E\,to\,PV}Q_{PV\,to\,E}$, etc.) and therefore $\det(M - rI) > 0$ which is a contradiction. Therefore, we proved that $Q_{E\,to\,E} - 1 > 1 + 2\alpha$ and it follows that $\Im(\nu_2) > 0$.

Appendix B

We show that for any choice of $Q_{SOM \to E}$ and $Q_{SOM \to PV}$, the matrix F possesses one real eigenvalue and a pair of complex conjugate eigenvalues. Eigenvalues of F are the roots of its characteristic polynomial and therefore are the solutions of $\lambda^3 + 2\lambda^2 + (6 + 4Q_{SOM \to E})\lambda + (20Q_{SOM \to E} - 20Q_{SOM \to PV} + 5) = 0$.

Let us denote $6 + 4Q_{SOM\rightarrow E}$ and $20Q_{SOM\rightarrow E} - 20Q_{SOM\rightarrow PV} + 5$ by g and h, respectively. The cubic discriminant of the polynomial is then $\Delta = -4g^3 + 4g^2 + (36g - 32)h - 27h^2$. View Δ as a quadratic in h: $\Delta(h) = -27h^2 + (36g - 32)h + (4g^3 - 4g^2)$. The discriminant of $\Delta(h)$ (as a quadratic in h) is $D_h = -16(3g - 4)^3$. We know that $g = 6 + 4Q_{SOM\rightarrow E}$ and therefore g is always larger than 6 resulting in $D_h < 0$. Since the coefficient of h^2 in $\Delta(h)$ is $-27 < 0$, the quadratic $\Delta(h)$ opens downward and - having negative discriminant - never meets the h axis. Thus $\Delta(h) < 0$, for all h and so we proved that the cubic discriminant of F is always smaller than 0. $\Delta < 0$ dictates that the characteristic polynomial of F has exactly one real and two non-real conjugate roots. Hence, we showed that no matter what $Q_{SOM\rightarrow E}$ and $Q_{SOM\rightarrow PV}$ are, F has exactly one real eigenvalue and a pair of complex conjugate eigenvalues.

Appendix C

Let us denote $Q_{PV\,to\,SOM}$ by x and so the characteristic polynomial of Y is $p(\lambda\,,\,x) = \lambda^3 + 2\lambda^2 + (22 - \frac{41}{20}x)\lambda - \frac{59}{5}x + 44$. By reasoning like the one provided for matrix F (by analyzing the discriminant of the polynomial), we conclude the matrix Y has a pair of complex conjugate eigenvalues and a real eigenvalue. $p(-2, x) = -7.7x$ and $p(0, x) = -\frac{59}{5}x + 44$ and therefore for any x ($0 \leq x \leq \frac{220}{59}$), we have $p(-2, x) \leq 0$ and $p(0, x) \geq 0$. Hence, by the intermediate value theorem, it turns out that the real eigenvalue of Y is in $[-2, 0]$. Let us denote the real eigenvalue of Y by $r(x)$. We have $p(r(x), x) = 0$ and by differentiating both side we derive

$$\frac{dr(x)}{dx} = -\frac{\partial_x p}{\partial_\lambda p}\bigg|_{\lambda=r(x)} = \frac{\frac{41}{20}r(x) + \frac{59}{5}}{3(r(x))^2 + 4r(x) + (22 - \frac{41}{20}x)}.$$

We already know that $0 \leq x \leq \frac{220}{59}$ and $0 \leq r \leq \frac{220}{59}$ and therefore both the numerator and denominator are positive and therefore $r(x)$ is a strictly increasing function of x. On the other hand, the trace of Y equals the sum of its eigenvalues and so $\alpha(x) = -1 - \frac{r(x)}{2}$ where $\alpha(x)$ is the real part of the complex conjugate eigenvalues. Consequently, it follows that $\alpha(x)$ is negative and a strictly decreasing function of x.

Appendix D

Let us denote $Q_{SOM\,to\,SOM}$ by x and therefore the characteristic polynomial of L is $p(\lambda, x) = \lambda^3 + (x + 2)\lambda^2 + (x + 22)\lambda + (5x + 44)$. By reasoning using the discriminant of the polynomial similar to the one provided before one can show that the matrix L has a pair of complex conjugate eigenvalues and a real eigenvalue for any $x \geq 0$. Let's show the eigenvalues by $\alpha(x) \pm \beta(x)$ and $r(x)$. Implicit differentiation of p(r(x) , x) = 0 gives

$$\frac{dr(x)}{dx} = \frac{\partial_x p}{\partial_\lambda p}\bigg|_{\lambda=r(x)} = \frac{-(r(x))^2 - r(x) - 5}{3(r(x))^2 + 2(x + 2)r(x) + x + 22}.$$

For any $r(x)$, the numerator is always negative. On the other the denominator is

$$\partial_\lambda p|_{\lambda=r(x)} = \partial_\lambda((\lambda - r(x))(\lambda - \alpha(x) - i\beta(x))(\lambda - \alpha(x) + i\beta(x)))|_{\lambda=r(x)} = (r(x) - \alpha(x))^2 + \beta^2 > 0$$

and it follows that r'(x) is always negative. And so r(x) is a negative decreasing function of x, starting from r(0) = -2.

Equality of the summation of eigenvalues and the trace of L, results in $\alpha(x) = -\frac{x}{2} - r(x) - 1$. By similar analysis (omitted for brevity), one can show that $\alpha(x)$ starts from $a(0) = 0$, decreases strictly to its global minimum around -1.3 at around $x^* = 6$ and then it strictly increases to its limit, $\lim_{x\rightarrow\infty} = -\frac{1}{2}$.
